# Biodiversity of Potential Vectors of Rickettsiae and Epidemiological Mosaic of Spotted Fever in the State of Paraná, Brazil

**DOI:** 10.3389/fpubh.2021.577789

**Published:** 2021-03-12

**Authors:** Liliane Silva Durães, Karla Bitencourth, Frederico Rodrigues Ramalho, Mário Círio Nogueira, Emília de Carvalho Nunes, Gilberto Salles Gazêta

**Affiliations:** ^1^Programa de Pós-Graduação em Biodiversidade e Conservação da Natureza, Universidade Federal de Juiz de Fora, Juiz de Fora, Brazil; ^2^Laboratório de Referência Nacional em Vetores das Riquetsioses- Secretaria de Vigilância em Saúde/Ministério da Saúde, Instituto Oswaldo Cruz/Fundação Oswaldo Cruz, Rio de Janeiro, Brazil; ^3^Departamento de Saúde Coletiva, Faculdade de Medicina, Universidade Federal de Juiz de Fora, Juiz de Fora, Brazil

**Keywords:** eco-epidemiology, flea, public health, rickettsiosis, tick, tick-borne disease, zoonosis

## Abstract

Spotted Fever Rickettsioses (SFR) are diseases caused by bacteria of the genus *Rickettsia*, and are transmitted mainly by ticks. Its eco-epidemiological scenarios vary spatially, and may also vary over time due to environmental changes. It is the main disease transmitted by ticks to humans in Brazil, with the state of Paraná (PR) having the sixth highest number of notified incidences in the country. However, information is lacking regarding the SFR disease cycles at likely infection sites within PR. During case investigations or environmental surveillance in PR for SFR, 28,517 arthropods were collected, including species known or potentially involved in the SFR cycles, such as *Amblyomma sculptum, Amblyomma aureolatum, Amblyomma ovale, Amblyomma dubitatum, Amblyomma parkeri, Ctenocephalides felis felis*, and *Rhipicephalus sanguineus* sensu lato. From these *Rickettsia asembonensis, Rickettsia bellii, Rickettsia felis, Rickettsia parkeri* strain Atlantic Rainforest and *Candidatus* Rickettsia paranaensis were detected. Ectoparasite abundance was found to be related with specific hosts and collection environments. Rickettsiae circulation was observed for 48 municipalities, encompassing 16 Health Regions (HR). As for socio-demographic and assistance indicators, circulation occurred largely in the most urbanized HR, with a higher per capita Gross Domestic Product, lower Family Health Strategy coverage, and with a higher ratio of beds in the Unified Health System per thousand inhabitants. For environmental variables, circulation occurred predominantly in HR with a climatic classified as “subtropical with hot summers” (Cfa), and with forest type phytogeographic formations. In terms of land use, circulation was commonest in areas with agriculture, pasture and fields and forest cover. Rickettsiae were circulating in almost all hydrographic basins of PR state. The results of this study provide the first descriptive recognition of SFR in PR, as well as outlining its eco-epidemiological dynamics. These proved to be quite heterogeneous, and analyzed scenarios showed characteristics strongly-associated with the outbreaks, with cases presenting clinical variation in space, so illustrating the complexity of scenarios in PR state. Due to the diversity of the circumstances surrounding SFR infections in PR, public health initiatives are necessary to foster a better understanding of the dynamics and factors effecting vulnerability to SFR in this Brazilian state.

## Introduction

Rickettsioses are zoonosis transmitted by arthropod vectors, and are considered to be emerging or re-emerging in several regions of the world. Their bioagent are Gram-negative, obligately intracellular bacteria of the genus *Rickettsia*. *Rickettsia rickettsii*, in the Spotted Fever Group (SFG), is the causative agent of Brazilian Spotted Fever (BSF), which is considered the main human tick-borne disease in the country, due to its high lethality rates. Other SFG rickettsioses, mainly that caused by *Rickettsia parkeri* strain Atlantic Rainforest and *Rickettsia parkeri* sensu stricto, are considered secondary due to their milder clinical courses and lack of lethality (1–4). Within the dynamics of disease transmission, humans are accidental hosts, and the epidemic cycle develops from focal infections, and generally remains highly-localized. However, multiple SFG species can coexist in the same environment, so that several species of ticks that parasitize different mammals can become infected (1–3, 5). Thus, the Spotted Fever Rickettsioses (SFR) enzootic and epidemic cycle in Brazil can be quite complex, since there is a diversity of both infecting agents and a diversity of potential vectors. This results in a variety of potential eco-epidemiological scenarios.

Ticks can act as vectors and reservoirs of SFG rickettsiae. Maintenance of bacterial infection in such arthropods occurs through feeding on a rickettsemic host and transovarian and/or transtadial transmission. Once infected, transmission to vertebrates occurs when the ectoparasite feeds (1, 3, 6). Thus, rickettsial presence in a given area is related to the existence of species of ixodids susceptible to infection and vertebrates capable of sustaining the tick population. Both of these can vary over time and in space. Additionally, the SFR epidemic cycle can be influenced by the overlap of human activities with tick seasonality (2, 7, 8).

Currently four SFR epidemic scenarios are known in Brazil: [1] the BSF, whose bioagent is *R. rickettsii*, with *Amblyomma sculptum* as its vector in the Southeast and parts of the Southern region; [2] *Amblyomma aureolatum* vectoring in the metropolitan area of São Paulo (SP), in the Southeast region of Brazil; [3] *Amblyomma ovale* vectoring *R. parkeri* strain Atlantic Rainforest in Atlantic Forest fragments in parts of the South, Southeast and Northeast regions of the country; and [4] *Amblyomma tigrinum* infected with *R. parkeri* s.s. in the Pampa biome in the South region (2, 4, 9).

In Brazil, SFR cases are collated by the Sistema de Informação de Agravos de Notificação (SINAN; Notifiable Diseases Information System), which aims to collect, gather and disseminate data among the health surveillance network and other interested parties. In 2001, SFR was considered by the Brazilian Ministry of Health to be a compulsory notification disease and, in 2014, it was included in the list of immediate compulsory notification diseases (within 24 h) (10, 11). Currently, there are confirmed cases of SFR in 20 of the 27 federative units within the country, with the majority occurring in the Southeast (1,555/2,127) and South (520/2,127) regions. However, deaths are concentrated in the Southeast region (675/683), and all deaths in the South region (6/683) are recorded in the state of Paraná (PR) (12, 13).

In this context, PR was the last state in the Southern region of Brazil to report SFR, with the first human SFR case recorded only in 2006 (14). However, by 2019 there were already 47 confirmed cases across the state, and the data from SINAN (12) in the individual form of epidemiological investigation, from January 2006 to December 2017, show *R. rickettsii* as a possible bioagent of 8/435 reported cases. Accordingly, PR constitutes a relatively new area in the SFR transmission scenario within the country, having for years been a lacuna within confirmed rickettsia distributions between the states in the Southeast and South regions of Brazil. However, knowledge about the eco-epidemiological aspects of the disease in the state is still poorly-developed. Accordingly, the current study aimed to analyze the eco-epidemiological profile of SFR in PR, including identifying and mapping the occurrence of potential vector ectoparasites and infection by rickettsiae, and evaluating the sociodemographic, and environmental factors associated with cases of the disease in the state, as well as levels of relevant government programs.

## Materials and Methods

### Study Area

**Figure 1:**
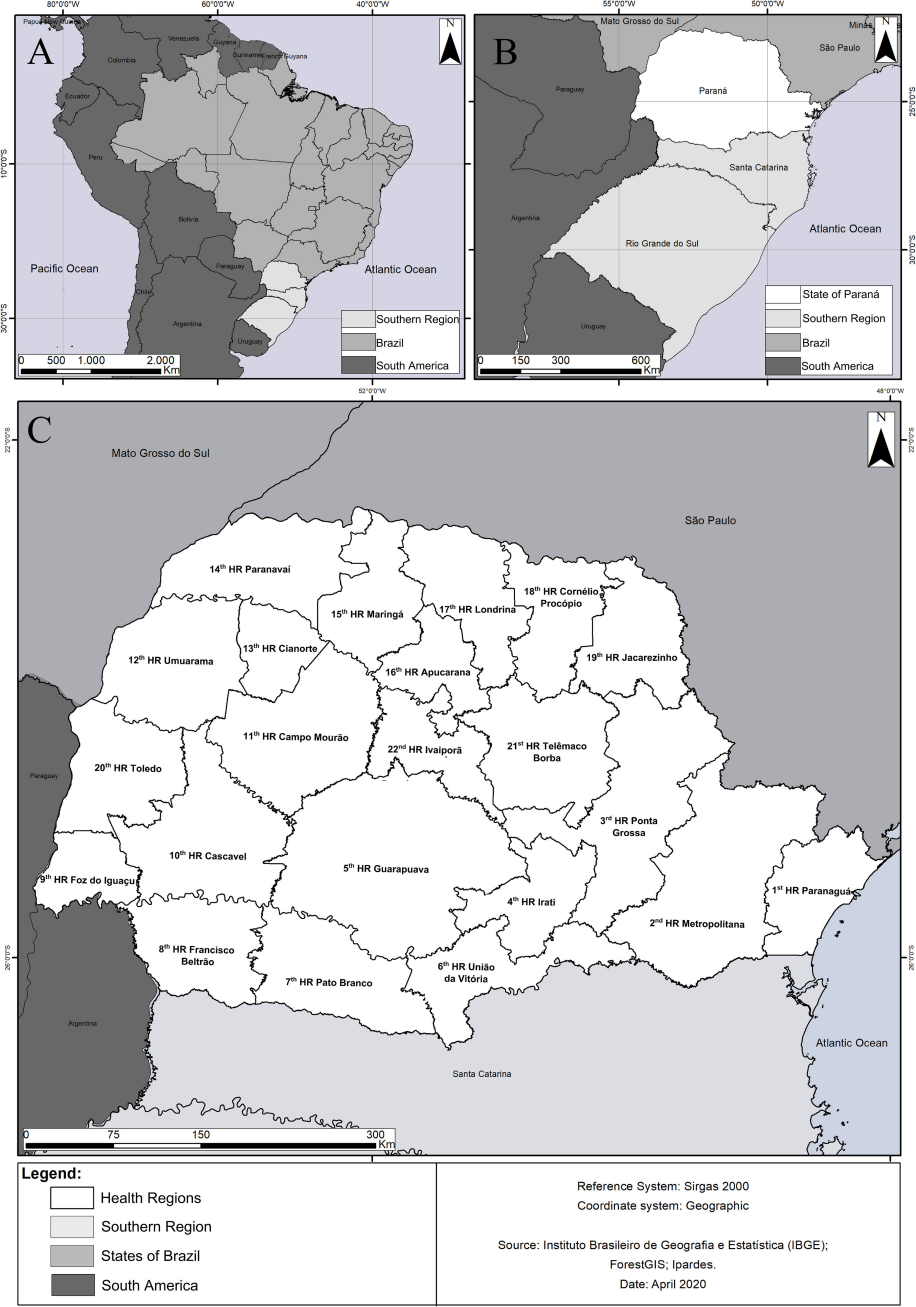
**(A)** Map of Latin America, highlighting the location of Brazil. **(B)** Southern Brazil, highlighting the state of Paraná. **(C)** Map of the state of Paraná, highlighting the Health Regions.

Paraná state, one of the 27 federative units in Brazil (Figure 1A), is located in the northern portion of the Southern region of the country (Figure 1B). Covering 199,305.236 km^2^, it is composed of 399 municipalities; these being divided between 22 Health Regions (HR) (Figure 1C). Due to its geographical position, the state lies at the intersection between climatic zones, subtropical with hot summer (Cfa), inserted in the Atlantic Forest biome, and temperate with mild summer (Cfb), occurring mostly in the Atlantic Forest biome, with a small portion in the Cerrado biome. According to the 2019 census, PR has a population of 11,433,957 inhabitants, with a demographic density of 52.40 inhabitants/km^2^. It has a Human Development Index (HDI) of 0.749, highest the fifth best in the country (15).

### Epidemiological Data

This is a descriptive and exploratory eco-epidemiological study of the notified and confirmed cases of the relationship between exposure and SFR outcome. The analytical units were the municipalities, and the HR in PR, with a time horizon of 11 years (January/2006–December/2017).

In Brazil, every suspected case of SFR requires compulsory notification and investigation, as it is a serious disease (16). Cases were classified as “suspected” whenever the individual presented sudden onset fever, headache, myalgia, and/or had a history of tick bites, and/or contact with domestic and/or wild animals, and/or had been to SFR known transmission areas in the last 15 days, and/or presented maculopapular rash or hemorrhagic manifestations (16). Cases were confirmed when signs, symptoms and epidemiological history matched the suspect case definition, and when infection with SFG rickettsiae was detected by serology (16).

Data were obtained from SINAN, using individual epidemiological investigation forms for notified cases, including notifications of suspected SFR cases (17), made available with protection of patient identities, and complying with regulated national ethical requirements (18). Data were filtered to identify duplications, inconsistencies and typos in the epidemiological records, with subsequent exclusion.

Each confirmed SFR case was georeferenced to municipality, which was then considered as a Probable Infection Locality (PIL). Analyzed outcome variable was the SFR cases notified and confirmed during the studied period. Subsequently, these data were grouped into: mild cases (without hospitalization and death), hospitalizations and deaths. Average annual rates of SFR incidence in HR were then estimated.

As exposure variables, we used environmental characteristics (climatic type, land use and cover and watersheds), as well as sociodemographic and health assistance information [degree of urbanization, Gross Domestic Product (GDP) *per capita*, coverage of the Family Health Strategy (FHS): *Estratégia de Saúde da Fam*í*lia* and ratio of beds registered in SUS (Unified Health System: *Sistema Único de Saúde*) per 1,000 inhabitants]. These variables were described using thematic maps, on which SFR cases were superimposed as points located in the centroids of the municipalities, the diameter of the points being proportional to the number of cases in each municipality. For the sociodemographic and assistance variables, maps were prepared with the distribution in quintiles, and obtained from the SUS Department of Informatics (DATASUS) website (http://tabnet.datasus.gov.br). For environmental variables, the categories available in the data obtained from the Instituto de Terras, Cartografia e Geologia do Paraná (Institute of Land, Cartography and Geology of Paraná) (http://www.itcg.pr.gov.br) website were used.

These analyses were made in the program R v. 3.6.1 using packages *Spdep, ClassInt* e *RColorBrewer* (https://www.r-project.org/), and the interface format *RStudio* v. 1.1.463 (https://rstudio.com/).

### Potential Vectors of SFR

This information was obtained from the database of the Laboratório de Referência Nacional em Vetores das Riquetsioses (LIRN; Laboratory of the National Reference of Rickettsial Vectors) of the Fundação Oswaldo Cruz (FIOCRUZ; Oswaldo Cruz Foundation), built from samples received and analyzed through the work of the Rede Nacional de Vigilância de Ambiente para Febre Maculosa e Outras Riquetsioses (National Network for Environmental Monitoring for Spotted Fever and other Rickettsial Diseases), from the Ministry of Health (19). This contains records of potential vector ectoparasites from 67 municipalities of PR, collected by the Surveillance teams of the Regional Health Services during investigations of cases and during environment surveillance for SFR, between the years 2013 and 2018. Collections were carried out in the environment (through flannel drag and active search) and on vertebrates (by active search). Collection sites were georeferenced from the HR to which the collection municipalities belonged.

Collected sampling units comprised of specimens from the same host or environment. Ectoparasites were screened and subjected to morphological identification using dichotomous keys (20–26). Reference specimens were deposited in the FIOCRUZ Coleção de Artrópodes Vetores Ápteros de Importância em Saúde das Comunidades (CAVAISC; Collection of Wingless Arthropod Vectors of Community Health Importance) (Supplementary Table 1), while the remaining specimens (28.276/28.517) were subjected to molecular analysis, resulting in 953 samples. In which, adult specimens were analyzed singletons or in pool of two and, non-adult specimens were pooled according to developmental stage (3–50 larvae and 1–20 nymphs).

Initially, the samples were subjected to total DNA extraction using the salt extraction technique of Aljanabi and Martinez (27), and PCR screening for rickettsia genes: *glt*A [CS2-78/CS2-323 (28), CS4-239/CS4-1069 (29)], *omp*A [190.70p/190.602n (30)], *htr*A [nested, 1st round 17k-5/17k-3 (29), 2nd round 17Kd1/17Kd2 (31)] and *omp*B [120-M59/120-807 (32)]. In all reactions, *R. rickettsii* DNA was used as a positive control and as a negative control, DNase and RNase-free Milli-Q water were used. As the function of the LIRN is to provide results indicating rickettsiae presence or absence in the analyzed ectoparasites, only part of the samples that amplified PCR products of the expected size were purified using the Wizard® SV Gel and PCR Clean-up System Kit (Promega, Corp., Madison, WI, USA), and sequenced automatically in both directions in an ABI 3730 xl Genetic Analyzer (Applied Biosystems, Inc., Carlsbad, CA, USA). Sequences were edited manually using the ChromasPro 1.5 program (Technelysium Pty Ltd., Tewantin, Qld, Australia). Sequences obtained [Genbank accession numbers MF175766, MF175773-4, MH194354, MH194360, MK252665-6, MK252669, MK252671-2, MK252676, MK252678-9, MK252682, MT311216-7, (33–36)] were automatically aligned with samples from GenBank, using the ClustalW algorithm (37). Phylogenetic inference was then performed using maximum likelihood in PhyML 3.0 (38), using the evolutionary model GTR + G indicated by MEGA 6.0 (39). Statistical support values for internal branches were estimated with the aLRT test (approximate likelihood-ratio test) with 1,000 replicates (40).

## Results

**Figure 2:**
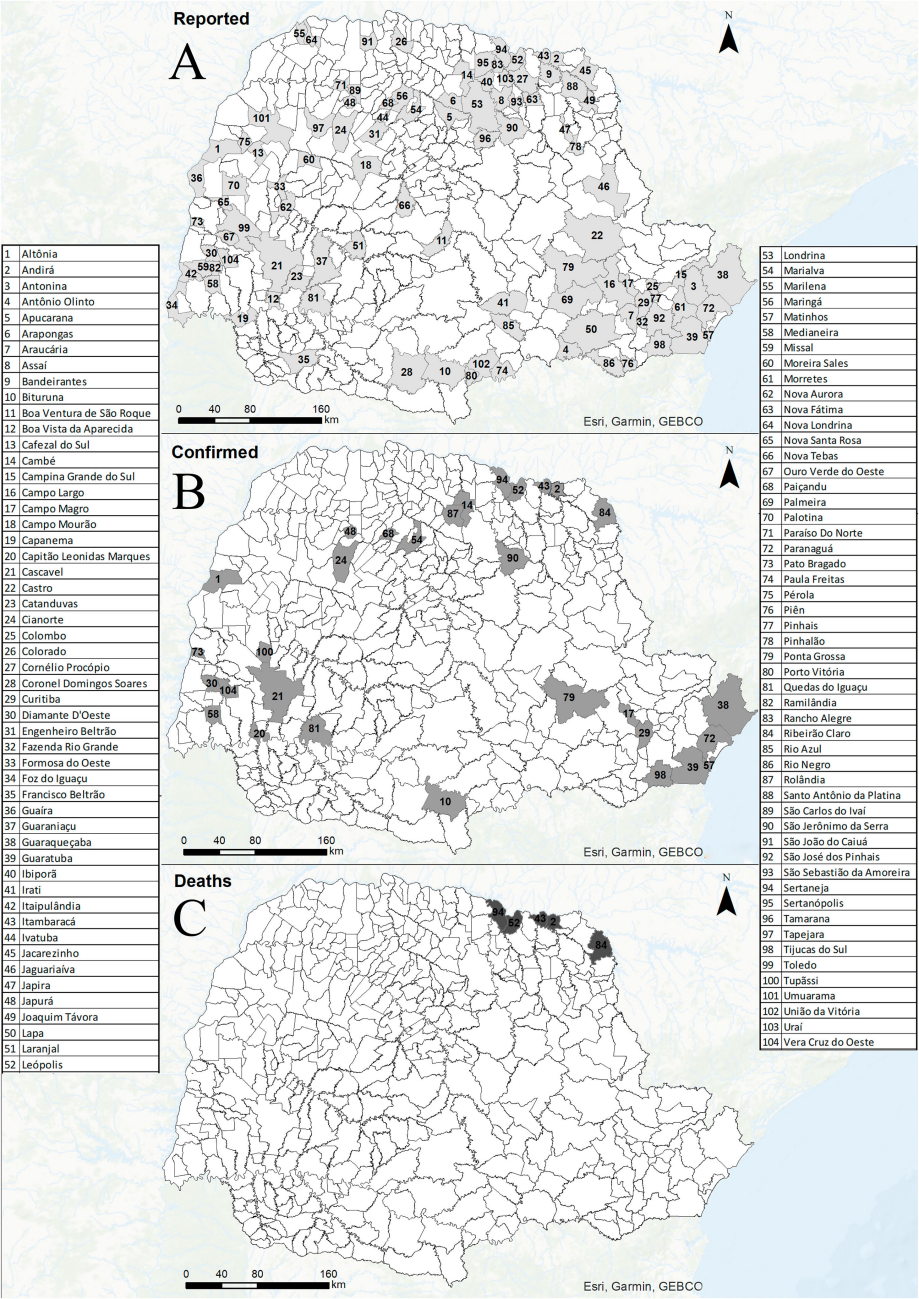
Reported cases **(A)**, confirmed cases **(B)**, and deaths **(C)** from Spotted Fever Rickettsioses in municipalities of Paraná state-Brazil, from 2006 to 2017.

Between 2006 and 2017, 437 suspected SFR cases were reported in PR, of which two were duplicates. Cases were reported from 25.10% (100/399) of state municipalities (Figure 2A). Of the total valid notifications, 98.20% (427/435) were from PR residents, 0.90% (4/435) from the state of Santa Catarina, 0.45% (2/435) from Mato Grosso state, and 0.45% (2/435) in another country (Paraguay).

**Figure 3:**
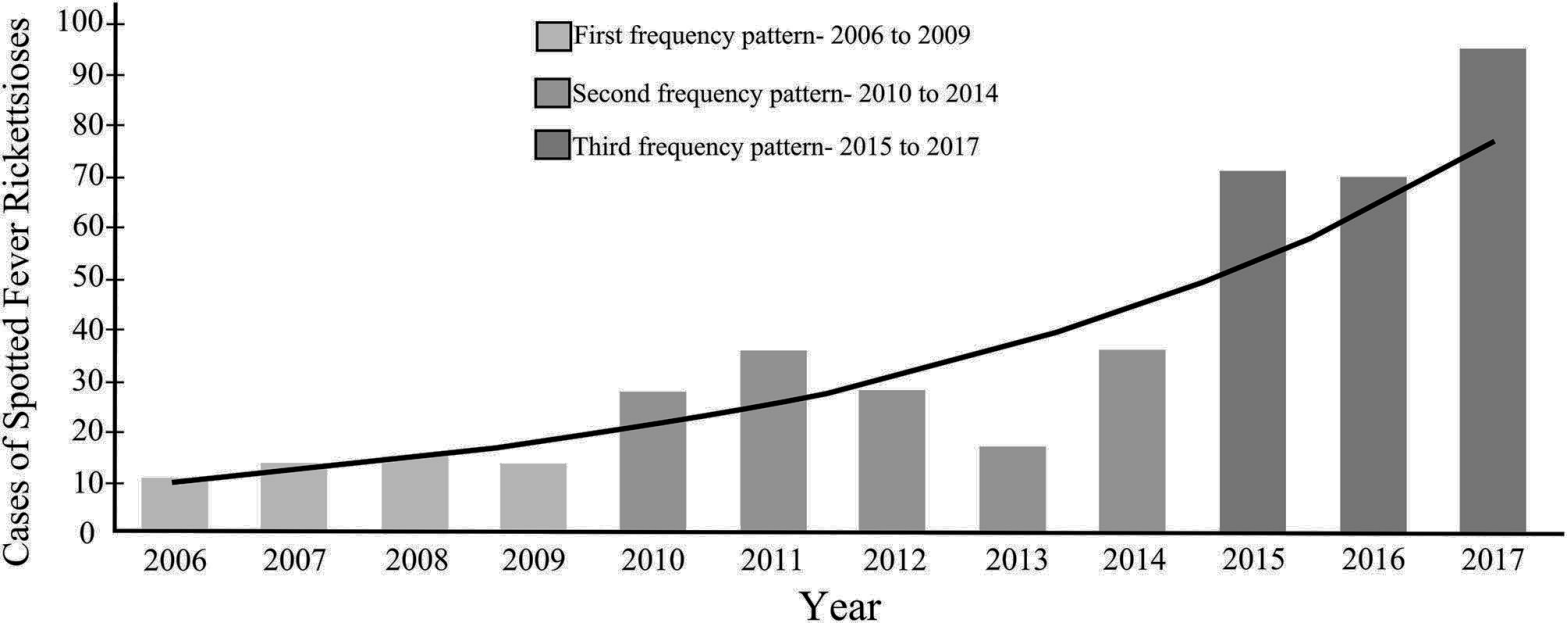
Number of reported cases of Spotted Fever Rickettsioses per year in the state of Paraná-Brazil, from 2006 to 2017.

**Figure 4:**
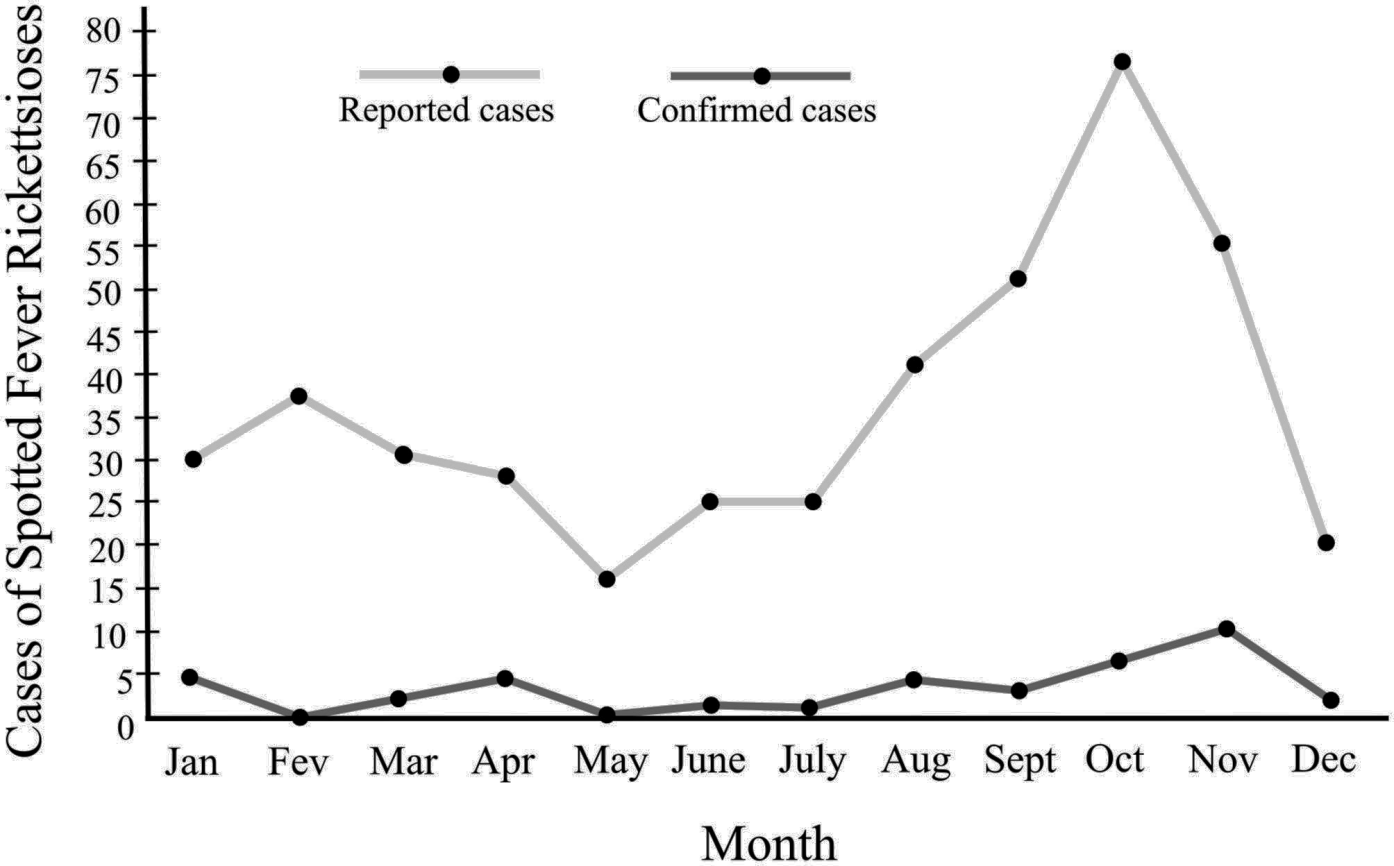
Temporal distribution of reported and confirmed cases of Spotted Fever Rickettsioses in the state of Paraná-Brazil, from 2006 to 2017.

Three inter-annual blocks in notifications frequency could be distinguished: 2006 to 2009, 2010 to 2014, and 2015 to 2017 (Figure 3). There were also within-year fluctuations: from January to April there was a constant number of notifications, with a fall in May, followed by an increase in June, a peak in October, and then a fall lasting until December (Figure 4). The time interval between onset of the first symptoms until notification was 7 days for most individuals.

The 2nd HR (Metropolitana) had the highest number of notifications (104/435: 23.90% of total), followed by the 18th HR (Cornélio Procópio) with (42/435: 9.65%) (Figure 1C). Reported individuals had an average age of 30.31 ± 19.67 years, with 62.99% (274/435) being male and 37.01% (161/435) female, of which 2.48% (4/161) were pregnant. Broken down by declared race, the sample had a predominance of whites (342/435: 78.62%), and 14.94% (65/435) being either black or *pardo* (mixed race). In terms of educational levels, the majority (32.88%: 143/435) had elementary school (incomplete/complete), 20.92% (91/435) high school (incomplete/complete), 13.10% (57/435) higher education (incomplete/complete), 1.38% (6/435) were illiterate. In 31.72% (138/435) of the cases the field had not been completed.

Of the competed notifications (435), 38 were noted as confirmed cases, according to the criteria established by the Ministry of Health (16). Of these, one case was excluded from the analysis because the PIL was located in another state (SP), so that 37 cases were analyzed (23 males, and 14 females: none of whom were pregnant) (Figure 2B). Confirmed notification rates ranged from 0.23 per 100,000 inhabitants in the 13th HR (Cianorte) to zero in nine other HR (Supplementary Table 2). For confirmation criteria, 81.08% (30/37) of the cases were confirmed by laboratory tests, 13.51% (5/37) by epidemiological clinical means, and in 5.41% (2/37) of the cases this field has not been completed.

For the PIL, 78.38% (29/37) were indigenous to the municipality of residence. As for PIL type, 70.27% (26/37) of individuals became infected in rural areas, 16.22% (6/37) in urban areas, 10.81% (4/37) in peri-urban areas, while for 2.70% (1/37) this field was not completed. Regarding the infesting environment, 35.13% (13/37) were infected in the home, 35.13% (13/37) in a leisure environment, 18.92% (7/37) in a workplace, 2.71% (1/37) in other environments, 5.40% (2/37) in an unknown environment, and on 2.71% (1/37) of cases this field was not completed.

Among the risk exposures of confirmed cases, 86.49% (32/37) reported contact with ticks. This was followed by 54.05% (20/37) who had contact with dogs and cats, equines 24.32% (9/37), cattle 21.62% (8/37) and capybaras 5.40% (2/37). Those who visited environments with forests, rivers, waterfalls and had a confirmed diagnosis were 75.67% (28/37).

The distribution of the 37 confirmed cases, according to first symptoms detection month, fluctuation across the year: a decline in February, with a subsequent increase until April, followed by absence of cases in May, and an increase from June to October there, a peak in November, and from there on, a decrease (Figure 4).

**Figure 5:**
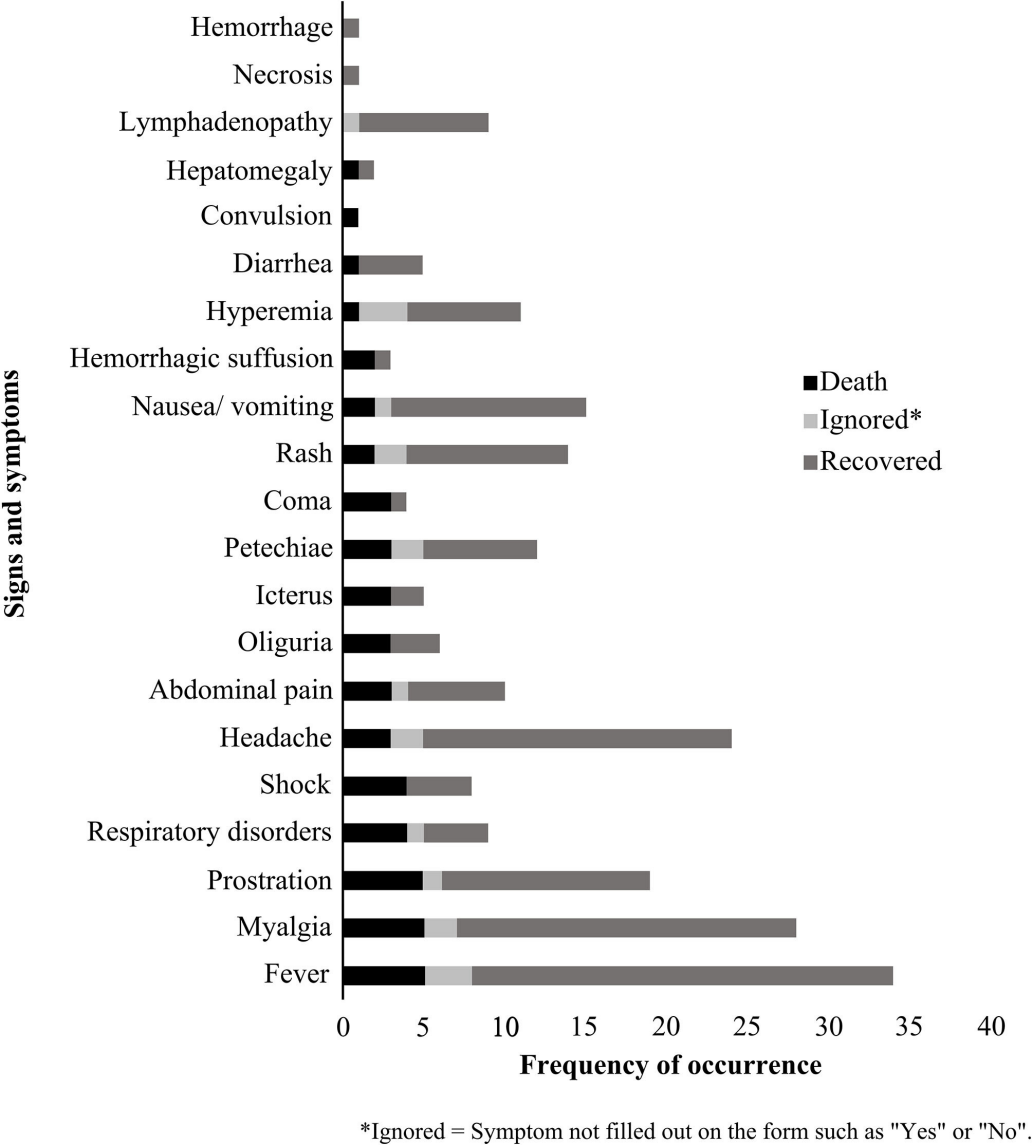
Main Spotted Fever Rickettsioses signs and symptoms reported to the Notifiable Diseases Information System, between 2006 and 2017 in the state of Paraná-Brazil.

The signs and symptoms most frequently reported in the 37 confirmed cases were: fever 91.89% (34/37), myalgia 75.67% (28/37) and headache 64.86% (24/37) (Figure 5). Mild cases (without hospitalization and death) occurred in 48.65% (18/37) of cases, with 51.35% (19/37) requiring hospitalization. For the outcome of confirmed cases: 75.67% (28/37) recovered, while 13.51% (5/37) culminated in death from SFR.

Of the five deaths, three were women and two were men, all living in urban areas. As for PIL characteristics, one (woman) was from a home environment in a peri-urban area and four (two women and two men) from rural areas, two of which (a woman and a man) were related to leisure activities, one (man) to the work routine and one (woman) with field unfilled. In terms of symptoms, all reported fever, myalgia and prostration; while four individuals (two women and two men) had shock and respiratory changes; and two (a woman and a man) had a rash. The median time of disease progression (date of onset of the first symptoms to death) was 13 days (ranging from 6 to 16 days), and the mean time for notification of deaths (date of onset of symptoms and notification) was 15.6 days (ranging from 8 to 28 days).

**Figure 6:**
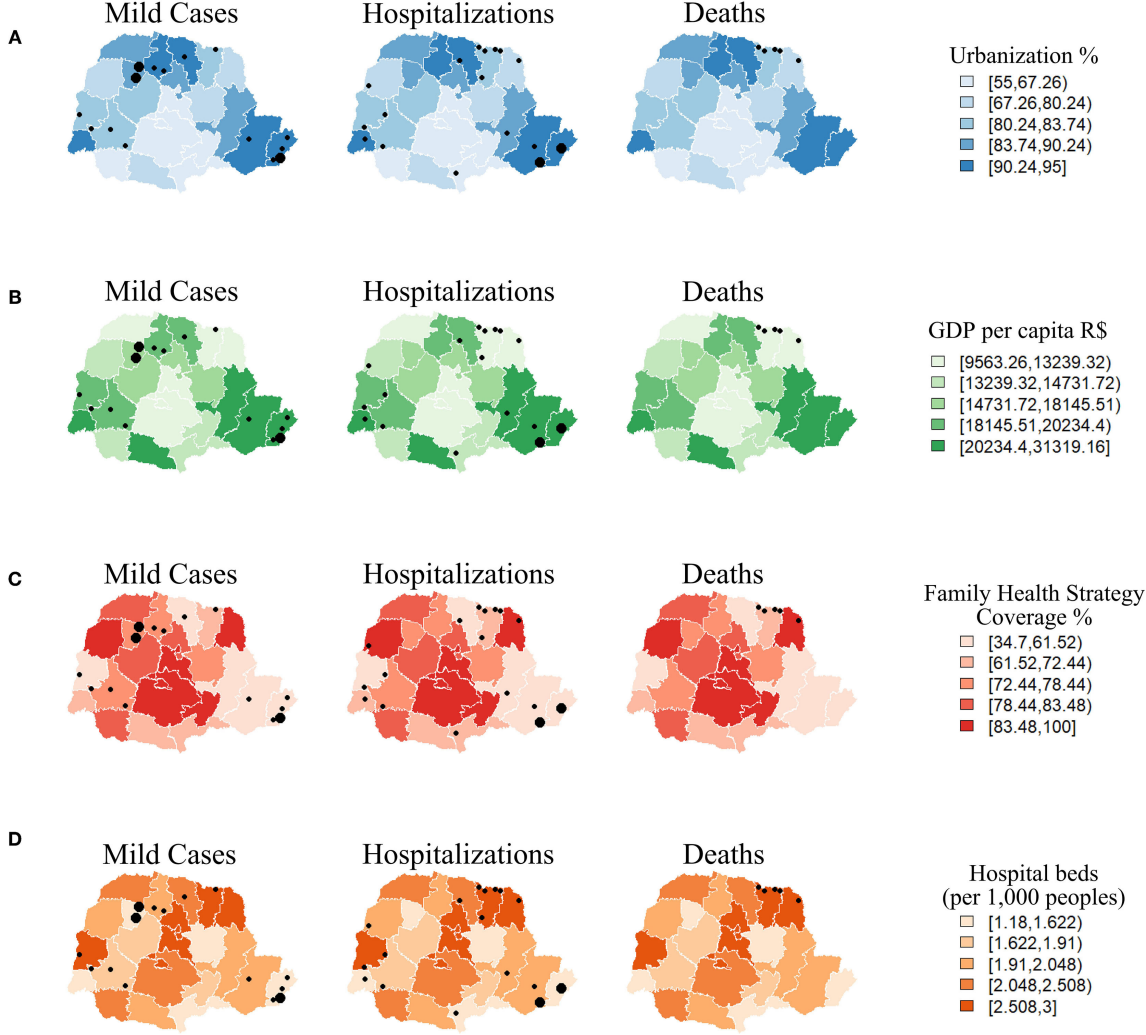
Distribution of Spotted Fever Rickettsioses cases in the state of Paraná-Brazil, from 2006 to 2017, according to socio-demographic and care indicators [**(A)** Urbanization, **(B)** Gross Domestic Product, **(C)** Family Health and **(D)** Hospital beds].

Mild SFR cases predominated in the most urbanized HR, with higher GDP per capita and FHS coverage ranging from 34.7 to 77.0% (Figures 6A–C; Supplementary Table 2). The most serious cases, which evolved to death, were concentrated in two HR situated in the northeast of the state, 18th HR (Cornélio Procópio: 4 deaths) and 19th HR (Jacarezinho: 1 death), both in the North Pioneiro mesoregion, which are among the less urbanized health regions, with lower GDP per capita and higher ratio of SUS beds per 1,000 inhabitants. The other cases of hospitalization, which did not progress to death, had a sparse distribution within the state (Figure 6; Supplementary Table 2).

**Figure 7:**
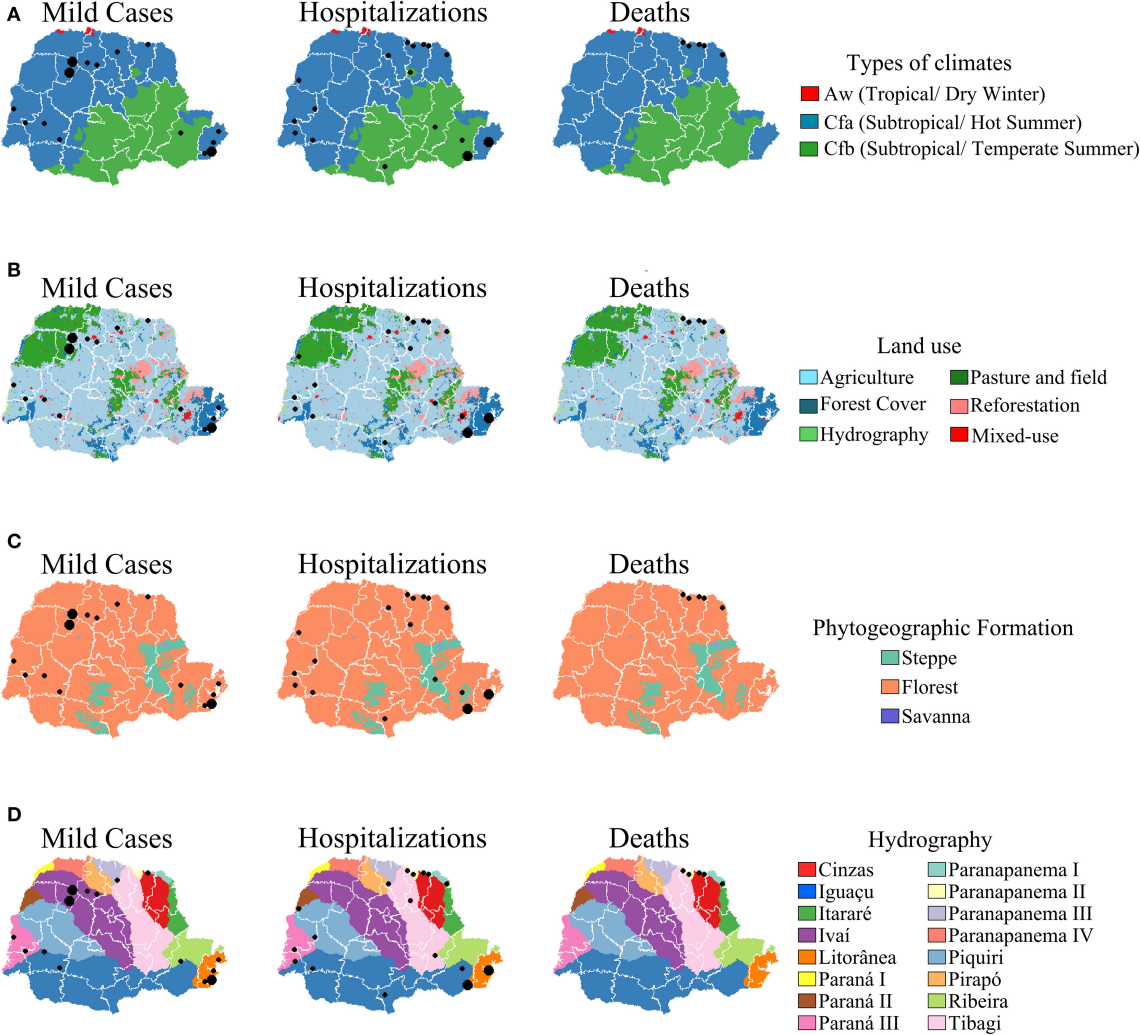
Distribution of Spotted Fever Rickettsioses cases in the state of Paraná-Brazil, from 2006 to 2017, according to environmental indicators. [**(A)** Types of climates, **(B)** Land use. **(C)** Phytogeography formation and **(D)** Hydrography].

Most SFR cases were recorded in Cfa climatic type areas with forest type vegetation (Figures 7A,C). For land use, mild cases and hospitalizations were located in areas with agriculture, pasture-and-fields and forest cover, but all deaths occurred in agricultural areas (Figure 7B). The Paranapanema I and II basins had most cases of death, while mild cases and hospitalizations occurred in a number of hydrographic basins within the state (Figure 7D).

The collections of ectoparasites occurred predominantly in the environment, while in the hosts, the following were sampled: asses (*Equus asinus*), cats (*Felis catus*), cows (*Bos taurus*), dogs (*Canis familiaris*), horses (*Equus caballus*), humans (*Homo sapiens*), pigs (*Sus scrofa*) and rodents (*Akodon* spp., *Hydrochoerus hydrochaeris* and *Oxymycterus nasutus*).

A total of 28,517 arthropods were collected, including samples of ticks, fleas, mites and lice. However, some immature specimens were only identified up to genus (*Amblyomma* and *Ixodes*) due to the lack of specific morphological identification (Table 1).

**Table 1:** Arthropods collected, from 2013 to 2018, in the state of Paraná, Brazil.

**Arthropods**	**Larva**	**Nymph**	**Male**	**Female**	**Total**
*Amblyomma aureolatum*		2	27	52	81
*Amblyomma dubitatum*		7,741	669	555	8,965
*Amblyomma incisum*		7			7
*Amblyomma longirostre*		1		3	4
*Amblyomma ovale*		1	27	8	36
*Amblyomma parkeri*		9			9
*Amblyomma sculptum*		608	84	91	783
*Amblyomma* sp.	12,748	16		7	12,771
*Amblyomma varium*		20			20
*Dermacentor nitens*	638	128	118	254	1,138
*Ixodes auritulus*		6			6
*Ixodes* sp.	31				31
*Rhipicephalus microplus*	2,929	74	126	374	3,503
*Rhipicephalus sanguineus* sensu lato	234	266	264	254	1,018
*Ctenocephalides felis felis*			40	90	130
*Polygenis roberti*				1	1
*Pulex irritans*			2		2
*Androlaelaps rotundus*				4	4
*Gigantolaelaps* sp.				4	4
*Gigantolaelaps wolffsohni*				3	3
*Haematopinus suis*				1	1
Total	16,580	8,879	1,357	1,701	28,517

Of the 67 municipalities in which collections took place, that with the highest species richness was Inajá (14th HR, Paranavaí) with six species: *Amblyomma dubitatum, A. sculptum, Ctenocephalides felis felis, Dermacentor nitens, Rhipicephalus microplus*, and *Rhipicephalus sanguineus* sensu lato (Supplementary Table 3).

From the total samples of ticks (930), fleas (20) and mites (3), submitted to PCR, were detected fragments of: 1- *glt*A in 172 tick samples (*A. aureolatum, A. dubitatum, Amblyomma longirostre, A. ovale, Amblyomma parkeri, A. sculptum, Amblyomma* sp., *D. nitens, R. microplus*, and *R. sanguineus* s.l.), and in 7 samples of *C. felis felis*; 2- *omp*A in 4 tick samples (*A. parkeri, A. sculptum, Ixodes* sp. and *R. microplus*) and in 4 *C. felis felis* samples; 3-*omp*B in 4 tick samples (*A. parkeri* and *R. microplus*) and 4-*htr*A in 4 tick samples (*A. parkeri* and *A. sculptum*).

**Figure 8:**
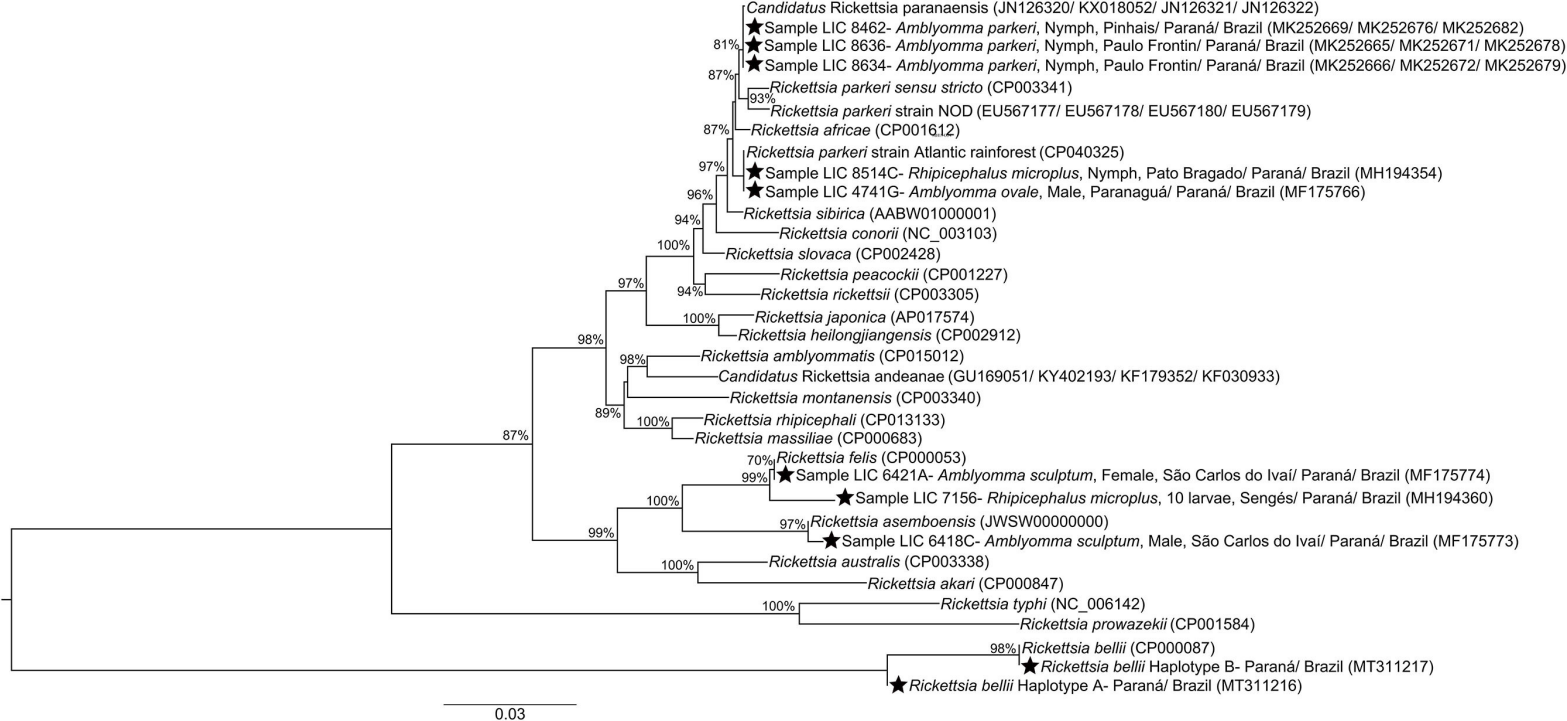
Concatenated phylogeny of rickettsia *gltA, htrA, ompA, and ompB* genes fragment (1201+ 370+ 494+ 822 bp) detected in analyzed ticks from state of Paraná-Brazil, inferred by Maximum Likelihood analysis with evolution model GTR+G. The numbers on the branches represent support values (70% cut-off). Stars indicate sequences obtained for Paraná. GenBank accession numbers are given in parentheses. Scale bar indicates nucleotide substitutions per site.

Of these samples, 91 were processed to investigate the species of rickettsia responsible for the infection in the ixodids, in which two haplotypes were identified, for the *glt*A gene, in *Rickettsia bellii* (hA in 70 samples and hB in 13) infecting *Amblyomma* sp. (larvae) and *A. dubitatum* (nymphs, females and males) in the municipalities of Andirá, Cambará, Itambaracá, Jacarezinho, Leópolis, Ribeirão Claro, Santa Mariana and Sertaneja; *R. parkeri* strain Atlantic Rainforest infecting *A. ovale* (*glt*A, male) in the municipality of Paranaguá (34) and *R. microplus* (*glt*A, *omp*A; nymph) in Pato Bragado (36); *Candidatus* Rickettsia paranaensis (*glt*A, *htr*A, *omp*A and *omp*B) in three samples of nymphs from *A. parkeri* from the municipalities of Pinhais and Paulo Frontin (35); *Rickettsia felis* in *A. sculptum* (*htr*A, female) (33) and *R. microplus* (*omp*B, larvae) (36) in the municipalities of São Carlos do Ivaí and Sengés, respectively; and *Rickettsia asembonensis* infecting *A. sculptum* (*htr*A, male) in São Carlos do Ivaí (33) (Figure 8; Supplementary Table 3).

**Figure 9:**
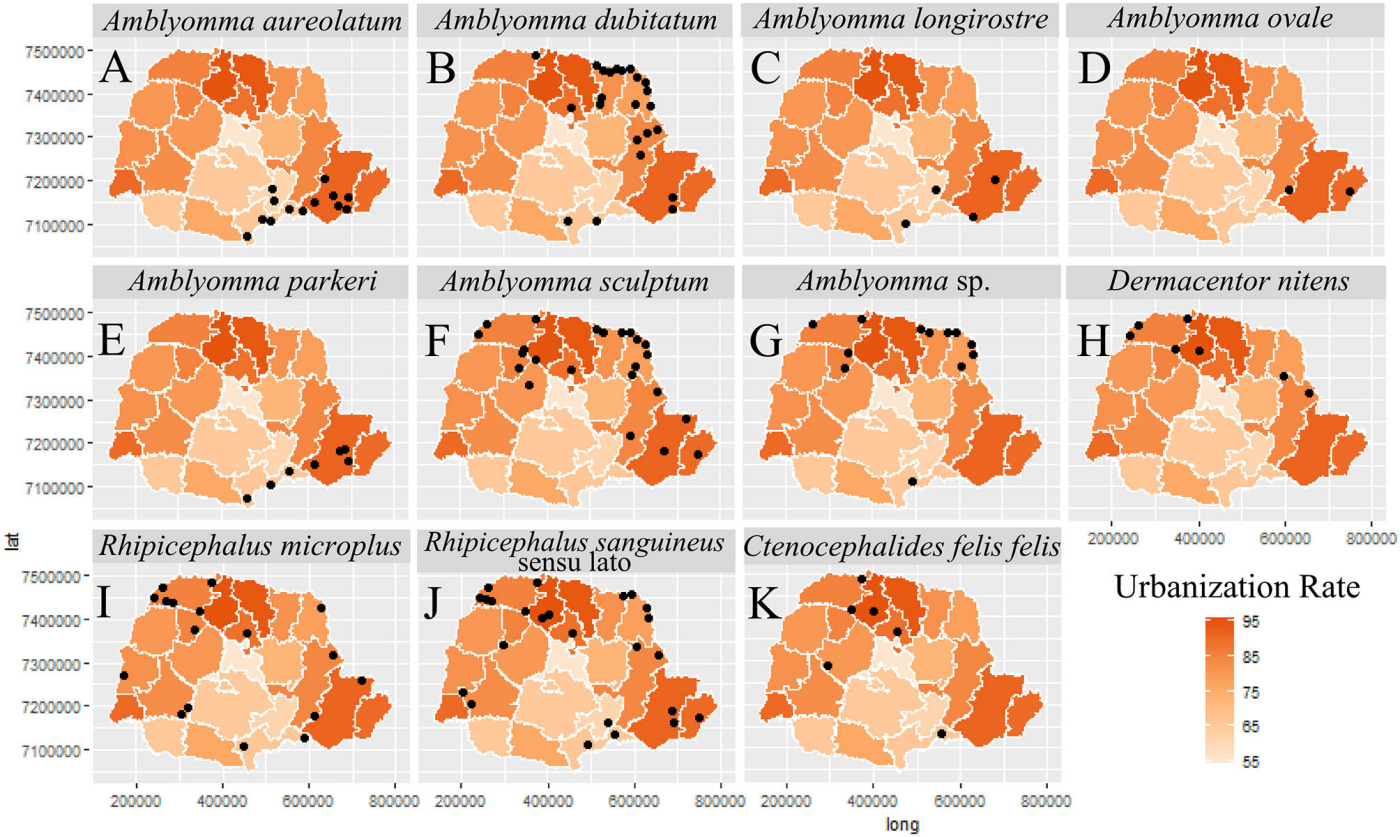
Distribution of potential vectors of Spotted Fever Rickettsioses in the state of Paraná-Brazil, from 2013 to 2018, according to urbanization rate. [**(A)** *Amblyomma aureolatum*, **(B)** *Amblyomma dubitatum*, **(C)** *Amblyomma longirostre*, **(D)** *Amblyomma ovale*, **(E)** *Amblyomma parkeri*, **(F)** *Amblyomma sculptum*, **(G)** *Amblyomma* sp., **(H)** *Dermacentor nitens*, **(I)** *Rhipicephalus microplus*, **(J)** *Rhipicephalus sanguineus* sensu lato and **(K)** *Ctenocephalides felis felis*].

**Figure 10:**
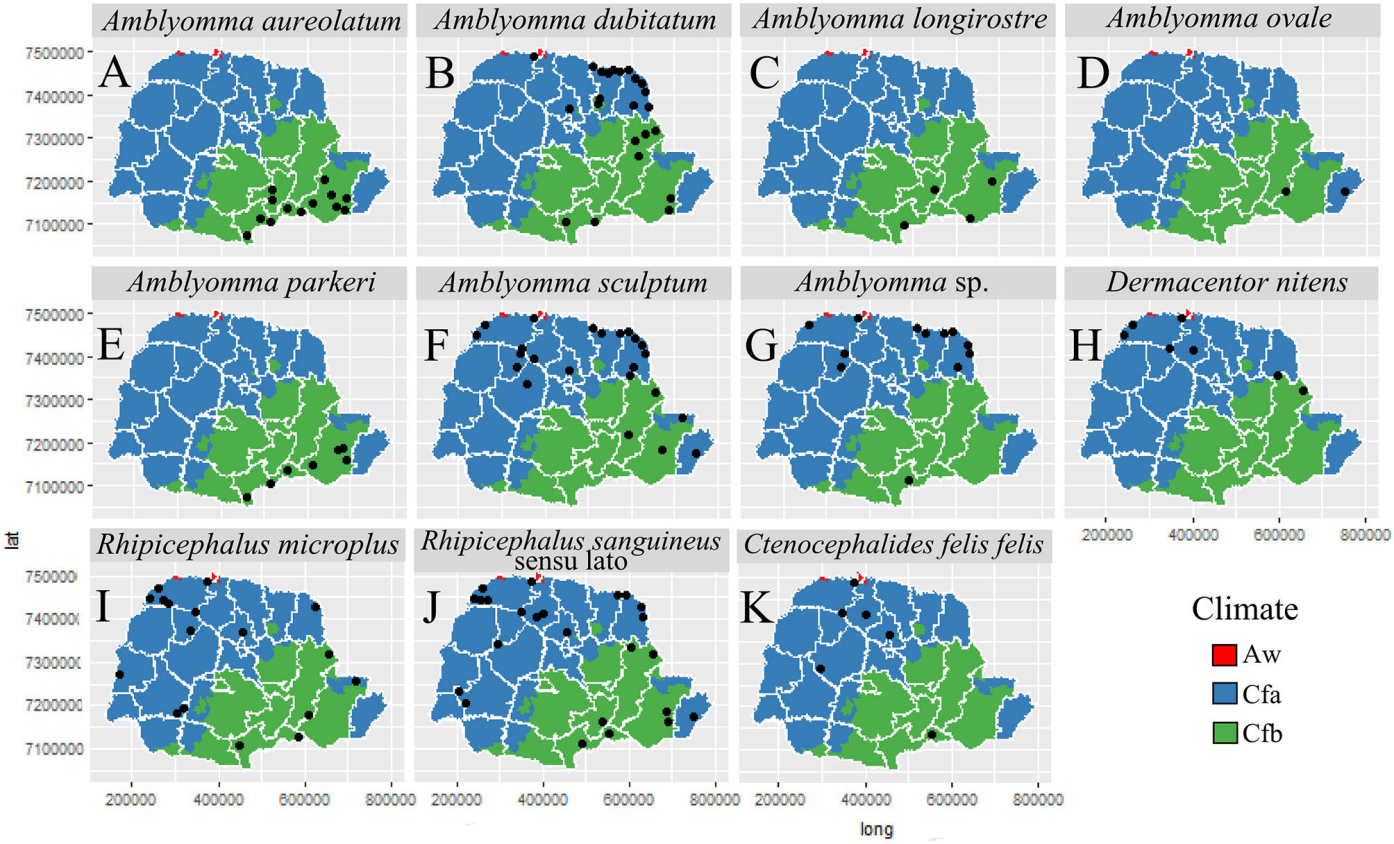
Distribution of potential vectors of Spotted Fever Rickettsioses in the state of Paraná-Brazil, from 2013 to 2018, according to types of climates. [**(A)** *Amblyomma aureolatum*, **(B)** *Amblyomma dubitatum*, **(C)** *Amblyomma longirostre*, **(D)** *Amblyomma ovale*, **(E)** *Amblyomma parkeri*, **(F)** *Amblyomma sculptum*, **(G)** *Amblyomma* sp., **(H)** *Dermacentor nitens*, **(I)** *Rhipicephalus microplus*, **(J)** *Rhipicephalus sanguineus* sensu lato and **(K)** *Ctenocephalides felis felis*].

**Figure 11:**
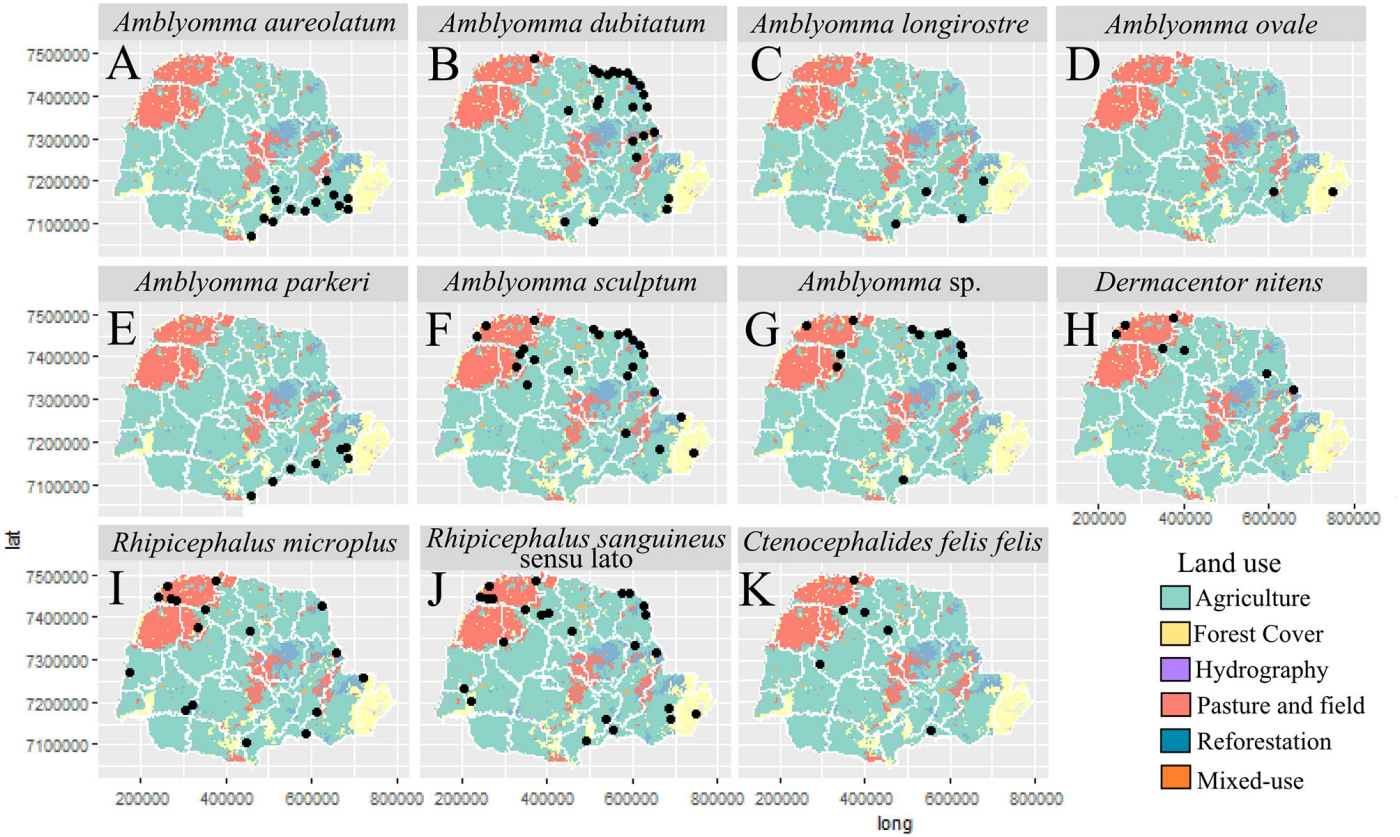
Distribution of potential vectors of Spotted Fever Rickettsioses in the state of Paraná-Brazil, from 2013 to 2018, according to land use. [**(A)** *Amblyomma aureolatum*, (**B)** *Amblyomma dubitatum*, **(C)** *Amblyomma longirostre*, **(D)** *Amblyomma ovale*, **(E)** *Amblyomma parkeri*, **(F)** *Amblyomma sculptum*, **(G)** *Amblyomma* sp., **(H)** *Dermacentor nitens*, **(I)** *Rhipicephalus microplus*, **(J)** *Rhipicephalus sanguineus* sensu lato and **(K)** *Ctenocephalides felis felis*].

**Figure 12:**
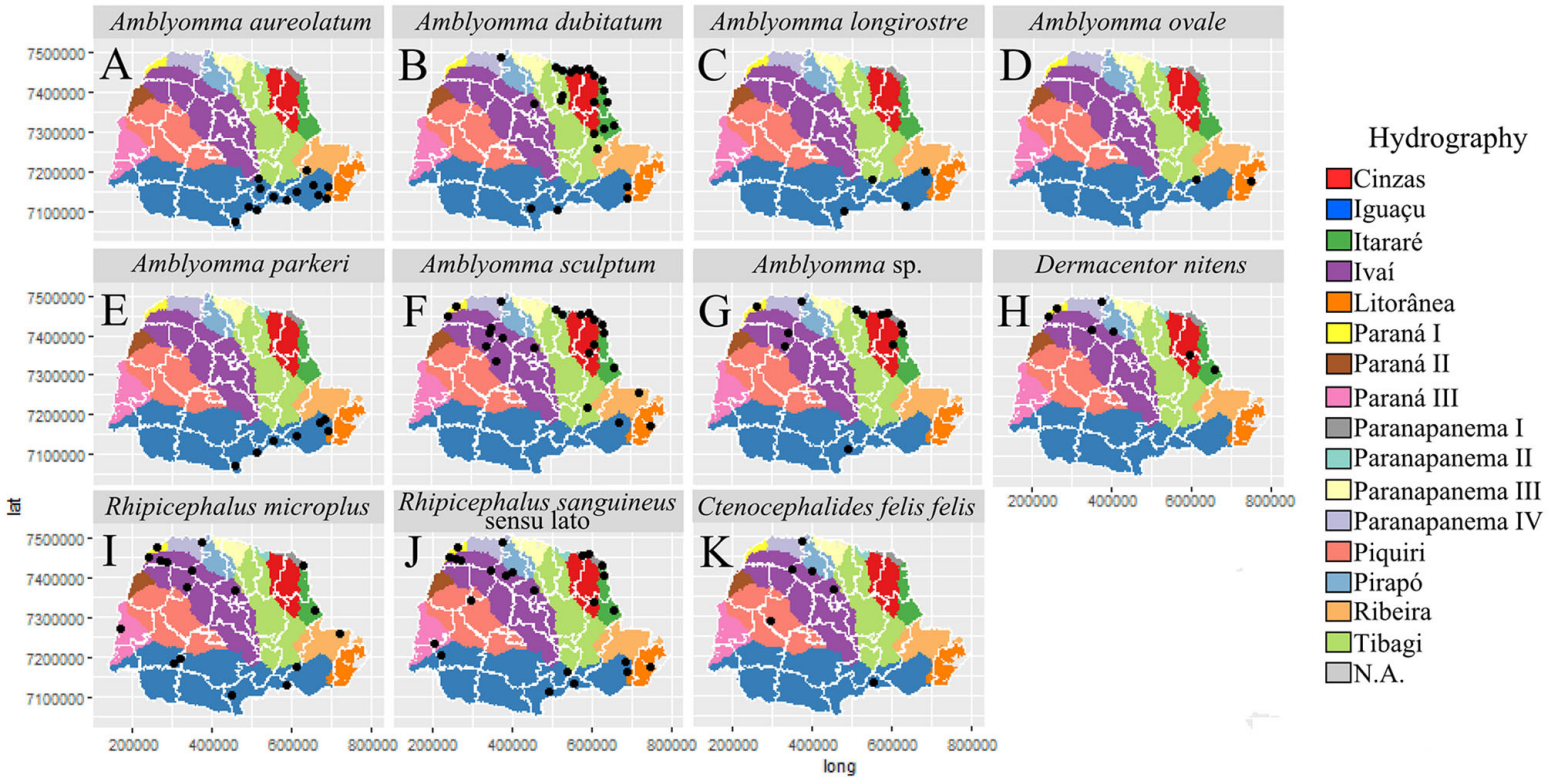
Distribution of potential vectors of Spotted Fever Rickettsioses in the state of Paraná-Brazil, from 2013 to 2018, according to hydrography. [**(A)** *Amblyomma aureolatum*, **(B)** *Amblyomma dubitatum*, **(C)** *Amblyomma longirostre*, **(D)** *Amblyomma ovale*, **(E)** *Amblyomma parkeri*, **(F)** *Amblyomma sculptum*, **(G)** *Amblyomma* sp., **(H)** *Dermacentor nitens*, **(I)** *Rhipicephalus microplus*, **(J)** *Rhipicephalus sanguineus* sensu lato and **(K)** *Ctenocephalides felis felis*].

In general, most of the potential vectors were collected in areas of HR with higher urbanization indexes (61.10–95.00%) (Figure 9). For environmental characteristics, the potential vectors were found in both climatic types (Cfa and Cfb) (Figure 10). However, *A. sculptum* was widely reported in the northern part of the state (the area with a Cfa type climate), where there are records of *R. rickettsii*-related SFR deaths (Figure 10F). The species *A. aureolatum, A. longirostre*, and *A. parkeri* dominated in the southeastern region of PR, in higher altitude areas and with Cfb climate (Figures 10A,C,E); *A. dubitatum* was recorded, predominantly, in the northeast in the Cfa area (Figure 10B); while the species *R. sanguineus* s.l., *R. microplus*, and *C. felis felis* occurred in both climatic types (Figures 10I–K). As for land use type, potential vectors were collected in areas with agriculture, pasture and field, and forest cover (Figure 11). Potential vectors were recorded in almost all river basins in the state (Figure 12).

**Figure 13:**
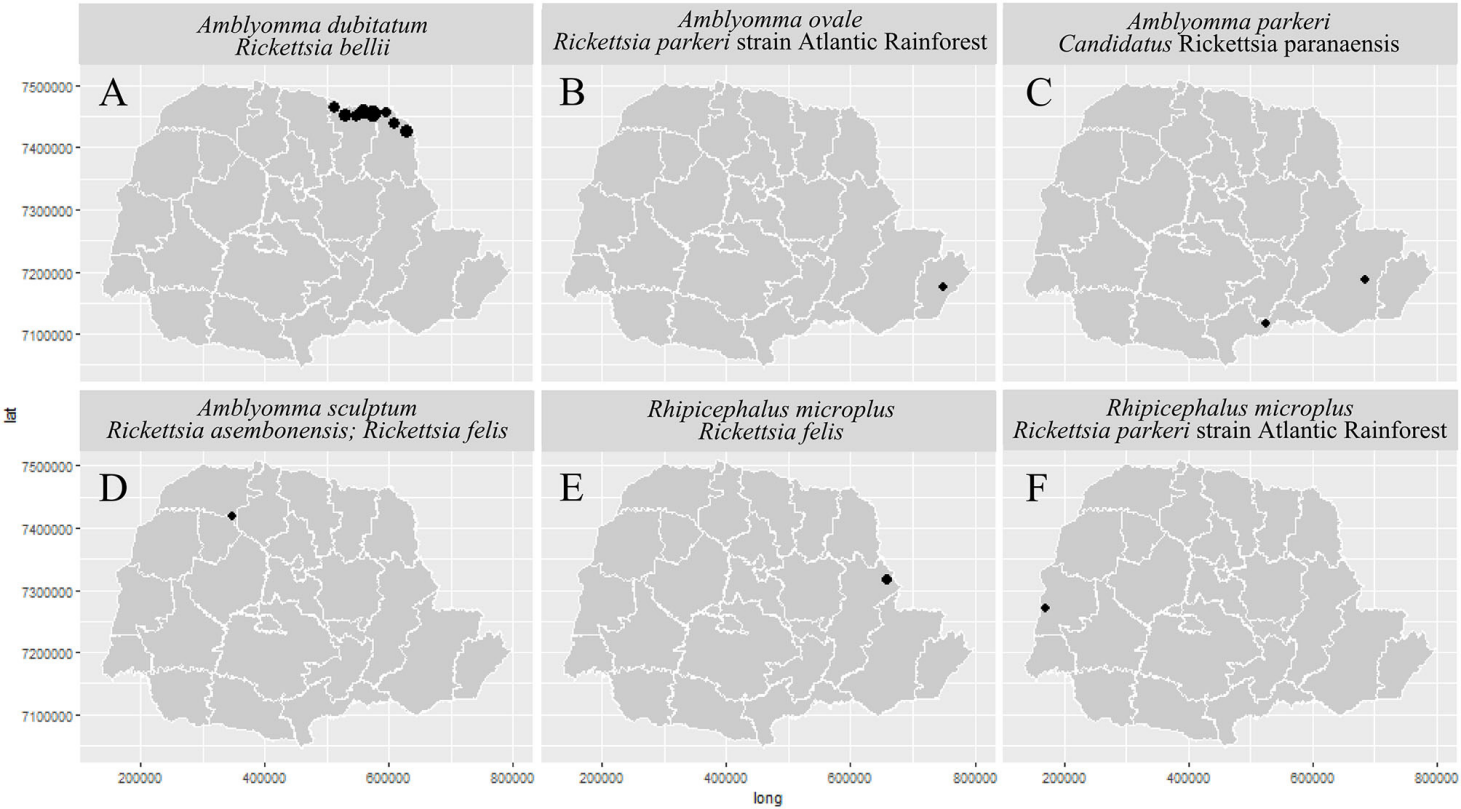
Distribution of *Rickettsia* spp. of Spotted Fever Rickettsioses in the state of Paraná-Brazil, from 2013 to 2018, according to Health Regions. [**(A)** *Amblyomma dubitatum/Rickettsia bellii*, **(B)** *Amblyomma ovale/Rickettsia parkeri* strain Atlantic Rainforest, **(C)** *Amblyomma parkeri/Candidatus* Rickettsia paranaensis, **(D)** *Amblyomma sculptum/Rickettsia asembonensis* and *Rickettsia felis*, **(E)** *Rhipicephalus microplus/Rickettsia felis*, and **(F)** *Rhipicephalus microplus/Rickettsia parkeri* strain Atlantic Rainforest].

Our results point to the occurrence of *A. ovale—*in the dog- with *R. parkeri* strain Atlantic Rainforest—on the coast (in preserved Atlantic Forest area); *A. parkeri—*in human-infected with *Ca*. R. paranaensis—in degraded Atlantic Forest; *A. dubitatum—*with *R. bellii—*associated with anthropization; *A. aureolatum—*in human- infected with *Rickettsia* sp.- in a forest edge area in an urbanized environment; *R. sanguineus* s.l.- in the dog- infected with *Rickettsia* sp. —in urbanized areas; *C. felis felis—*in a dog- with *Rickettsia* SFG—in anthropized areas. Even though there was no infection by *R. rickettsii* in the analyzed ticks, the occurrence of *A. sculptum—*its main vector, was recorded in anthropized areas were death had occurred (Figures 8, 13).

## Discussion

Ecopedemiological studies are essential for development of an accurate understanding of the different SFR scenarios [e.g., (41–43)], especially those which consider the complexity of transmission scenarios in the country, or analyze where knowledge is lacking (2, 4, 9, 34, 36, 44–50). This hinders a more contextualized analysis, that could provide the comparative results needed for effective public health action.

In PR, the first confirmed SFR case occurred in 2006 (14), resulting in a number of subsequent studies in the state (51–60). The majority of these have provided basic information concerning rickettsia circulation in a given area, with little direct attempt to study case association or use Surveillance System data. As a result, despite advances in rickettsioses knowledge elsewhere in Brazil, there has been little improvement in the understanding of its enzootic and epidemic cycle in PR. Likewise, epidemiological understanding of SFR in PR remains poorly developed, even though there was an increase in the number of notifications following the first case (Figure 3), signaling greater awareness of the health system for the detection of cases.

The results of the current study allowed us to provide the first description of SFR in PR, which, within a general context, has an eco-epidemiological profile close to that already studied for other endemic areas of Brazil, particularly that of the Southeast region, whose bioagent is *R. rickettsii* has an eco-epidemiological scenario involving capybara-*A. sculptum*-sugar cane crops, and in the metropolitan area of São Paulo (SP) dogs-*A. aureolatum*-Atlantic Rainforest fragments; and also *R. parkeri* strain Atlantic Rainforest in coastal and inland areas from the same region (dogs-*A. ovale*-Atlantic Rainforest). However, as the PIL of each confirmed case in PR was considered using a centroid of municipalities, urban areas were used to represent the cases. This can lead to inaccurate spatial representation, and the analysis of related eco-epidemiological variables. For example, using only the urban area as main point of a PIL could underrepresent the relevance of water courses, gallery forests and sugar cane crops to sustain capybaras (amplifier hosts) and ticks (vectors/reservoirs) in the eco-epidemiology of BSF.

Our results show similarities with a recent study of SFR in PR, where data on parasitism of humans by ticks and the distribution of cases and potential vectors were analyzed (43). There are resemblances with: the encounter of *A. sculptum* and *A. aureolatum* parasitizing humans (Supplementary Table 3); the encounter of *A. sculptum* and *A. dubitatum* in Norte Pioneiro (Figures 9–11); the geographical distribution of mild cases, hospitalizations and deaths (Figures 6, 7).

In this context, the analysis of the frequency of cases in PR shows the presence of three frequency patterns (2006–2009, 2010–2014, and 2015–2017) (Figure 3). These have also been detected in other studies (9, 42), and show a direct relationship between public health actions and increases in SFR notifications, especially once Ordinance 1,271 of the Ministry of Health, of June 6, 2014 (11), redefined the classification of the disease as once requiring immediate compulsory notification (within 24 h). Likewise, our results indicate that the temporal distribution of notified cases is greater between August (penultimate month of winter) and December (late spring and early summer), with a peak in October (second month of spring) (Figure 4). This is also the period with the highest number of confirmed cases (Figure 4). Thus, between the final third of winter and the beginning of summer, there is a higher incidence of SFR in PR, which has also been observed for other endemic areas of Brazil (9, 61, 62). However, we have to consider the diversity of SFR eco-epidemiological scenarios already present in PR, and that these are associated with different species and stages of ticks. Accordingly, in Norte Pioneiro, the region of the state in which all deaths are concentrated, it is likely that the circulation of *R. rickettsii* occurs associated with *A. sculptum* nymphs, as these are especially prevalent between the months of June to September (63, 64). This coincides with the peak of SFR cases in the country (64). At the same time, there is another scenario, occurring in the PR coastal region, which very likely involves the transmission of *R. parkeri* strain Atlantic Rainforest by adult stages of *A. ovale*, which are more frequent in November (65, 66). As a result, reducing the SFR morbidity coefficient in PR requires vector control, adopting strategies specific to the species involved in the epidemic cycle, both in terms of the environment and of vertebrate hosts.

Most of the confirmed cases were male, corroborating data from SFR epidemiology studies elsewhere in Brazil (9) and from the United States (CDC, 2015). Our study showed that the highest incidence of confirmed cases was for men and women living in urban areas, but who became infected in rural areas. Therefore, it is important to consider the PIL for a better understanding of the SFR infection scenario. Where, males generally acquired the etiologic agent during leisure activities in rural areas, which seems to indicate during visits to wooded areas, forests, rivers and waterfalls, while female individuals were infected during leisure activities (in the rural area) and conducting domestic tasks in the home environment (in a peri-urban area). This profile has also been observed in elsewhere in Brazil, especially in the Southeast region, the area with the largest number of recorded cases and deaths in the country (67). Likewise, people who self-identified as “white,” constituted the majority of PR SFR patients, a pattern which also seems to be common in other regions of the country (9). Most of the PR population is made up of individuals who declare themselves to be “white.” The prevalence of “whites” in the population of PR is probably due to the historical colonization patterns in southern Brazil, which was largely settled by Europeans (Italians, Germans and Poles) during the mid-19th century (68).

As is common in other states in Brazil (9), for most cases analyzed here, serology was used as the confirmatory criterion. However, this diagnostic method does not determine the species of bioagent, and the results must be interpreted within an epidemiological clinical context, since identification of the tick with which the patient was in contact with remains important when confirming SFR infection (42, 67, 69, 70). However, contact with capybaras, present in 5.40% (2/37) of the reports in this study, does not seem to be a relevant factor in SFR infections in PR, though they have been reported as so in RJ (42).

Across Brazil the SFR epidemiological scenario appears to be quite variable. Thus, in the Southeast, severe cases and deaths occur where PILs are mainly in rural or peri-urban areas of the Atlantic Forest and Cerrado biomes, with the epidemic cycle involving the transmission of *R. rickettsii* by *A. sculptum* (2, 9, 45, 71, 72). In such areas, capybaras and horses are the main vertebrate hosts of this tick; as capybaras behaving as amplifying hosts for *R. rickettsii*, they are most often involved in the circulation of infections in anthropized environments; while horses, in addition to maintaining tick populations, act as sentinel hosts, because of their prolonged antibody persistence (2, 9, 44, 45, 71, 73). Infections mainly of men engaged in work or leisure activities in such environments, notably in areas close to water or when working with crops, especially sugar cane (2, 9, 45, 71). Additionally, there has been an increase in the number of cases occurring in peri-urban and urban areas and involving women infected in domestic environments (9, 63). This scenario was already well-known from urban and peri-urban areas near Atlantic Forest fragments, where *A. aureolatum* infected with *R. rickettsii* reaches households and peri- household environment via dogs that get infested with the tick vector when they go into the woods. However, the involvement of *A. aureolatum* as *R. rickettsii* vector is only recognized for a single locus, in the metropolitan region of SP state (2, 9, 71, 74). Thus, it is likely that another tick species, capable of transmitting *R. rickettsii*, and present in urban and peri-urban environments, may contribute to this increase in cases. In this context, *R. sanguineus* s.l., a common species in an anthropic environment, involved in the transmission of different species of pathogenic rickettsiae, in various parts of the world (1), is capable of both transstadial and transovarial transmission of *R. rickettsii* (75). The species has already been detected infected with *R. rickettsii* in several regions of Brazil (76–78), and is a potential rickettsia vector for BSF in urban and peri-urban environments. This underscores the need for studies that increase our knowledge of the role of *R. sanguineus* s.l. in the *R. rickettsii* epidemic and enzootic cycles in these environments. This scenario cannot be ruled out for PR, where six samples of *R. sanguineus* s.l. infected with *Rickettsia* sp., were recorded. Of these, three came from Norte Pioneiro, a HR with confirmed SFR deaths (Supplementary Tables 2, 3).

Further, and pertinent to the SFR cycles, is the fact that, despite the presence of *A. aureolatum* infected with *Rickettsia* sp. in the metropolitan region of PR (Supplementary Table 3), this ixodid does not seem to participate in the disease cycle outside SP (2), as it is where there are studies carried out. Therefore, future studies should pay attention to this data and try to elucidate the possible participation of *A. aureolatum* in the epidemiological scenario of the disease in PR. Although we did not detect *R. rickettsii* in our study, which is not uncommon, due to the deleterious effects that this bacterium causes in the tick, even in endemic areas where serious cases and deaths associated with *R. rickettsii* are known to occur (74, 79, 80), this scenario resembles that reported for the north of PR, the part of the state where all deaths are concentrated (Figure 2C), and where *A. sculptum* was found in high frequency (Figure 10F). Likewise, both data from the present study, and those given by Oliveira et al. (81), point to PR SFR death PILs being commonest in rural areas (with the infection occurring during leisure and work activities), or in peri-urban areas, within a home environment, which agrees with an established BSF scenario (1, 2, 9). On the other hand, the high levels of *R. bellii* infection of *A. dubitatum*, reported mainly from the area where the five deaths in PR were concentrated, may be associated with a rickettsial interference (82, 83). The infection of arthropods by rickettsiae pathogenic to humans (e.g., *R. rickettsii*) can be regulated by infection of non-pathogenic rickettsiae (e.g., *R. bellii*). This is the result of competition for systemic colonization of ixodids, a process whereby primary infection by one rickettsia species, excludes ovarian infection by another species (84–86).

The epidemiological scenario of mild and moderate cases in Brazil occurs where the PIL is the rural or urban environment, in a forested area or an edge of the Atlantic Forest biome in the South, Southeast and Northeast regions of the country, especially those areas close to the coast, and the epidemic cycle involving transmission of *R. parkeri* strain Atlantic Rainforest by *A. ovale*. The human acquires the bioagent when it comes into contact with the infected tick in the forest, or when its domestic dogs, with free access to the forest, carry the infected *A. ovale* to a household and peri- household environment, where human parasitism could subsequently occur (1, 2, 47, 87). Such a scenario probably occurs in the coastal region and south of PR, where we found areas of forest close to the urban environment, and where *A. ovale* infected by *R. parkeri* strain Atlantic Rainforest was detected [Supplementary Table 3; Figure 13B, (34)]. Given the detection of *R. microplus* with this rickettsia in the western region of PR (20th HR-Toledo), this tick is potentially implicated in the SFR enzootic cycle and can be used to detect rickettsiae presence and circulation (36). A similar role could be developed for *D. nitens*, since larvae samples with *Rickettsia* sp. were found in northwestern PR (14th HR-Paranavaí) (42) (Supplementary Table 3). Although *R. microplus* and *D. nitens* show little anthropophilic behavior, and vectorial incrimination reports are currently lacking from the literature, these species can act as indicators of rickettsia circulation in the area. It is important to clarify that the encountered rickettsia DNA does not necessarily confirm the possibility and viability of infection.

Additionally, in the southern part of the state, *A. parkeri*, a tick of known anthropophilic capacity, has already been found infected by *Ca*. R. paranaensis, a species genotypically close to *R. parkeri, Rickettsia africae* and *Rickettsia sibirica*. All are known to be pathogenic, and produce mild cases, pointing that isolated cases linked to *Ca*. R. paranaensis could be detected and confirmed in the coming years (35) (Supplementary Table 3; Figures 8, 13C). Another possible scenario producing mild cases is one in which the PIL is in a rural environment, in a forest area or forest edge of the Pampa biome, in southern Brazil, where the epidemic cycle seems to involve transmission of *R. parkeri* s.s. by *A. tigrinum* (4). However, the absence of *A. tigrinum* in the collected tick samples allows us to rule out this scenario in the currently-studied area.

In Brazil, dogs have been indicated as important indicator animals for a regional SFR epidemiological status, due to their involvement in some rickettsia infection scenarios, directly or indirectly in epidemic and enzootic cycles, or in hosting ectoparasites or rickettsiae (76, 88–90), as well as the potential risk of transmission to humans, following conveyance of vectors into a domestic environment. Thus, the encounter of *C. felis felis*, collected from dogs, infected with rickettsia in northern PR (Supplementary Table 3) points to potential involvement of this arthropod and its vertebrate hosts in the rickettsiae enzootic cycle. In this context, the bacteria *R. felis* and *R. asembonensis*, genetically close, are reported from fleas and ticks collected from dogs. Even though its pathogenicity is the subject of controversy (91, 92), the bioagent of Flea-borne Spotted Fever, *R. felis*, has a wide geographic distribution, and is associated with mild cases of rickettsioses, with two confirmed cases in Brazil, based on serological data (93). This fact, associated with the absence of other confirmed cases in the country where this bacterium has been isolated, or has followed all the criteria necessary to define the disease causing bioagent, has resulted in the absence of consensus among researchers concerning these cases in Brazil, and the pathogenicity of *R. felis*. However, although detected in several species of hematophagous arthropods, including *A. sculptum* (33, 48) and *R. microplus* in PR (36) (Supplementary Table 3; Figures 8, 13D,E), most reports are associated with *Ctenocephalides* sp. fleas. Recently described, *R. asembonensis*, is a bacterium associated with domestic animals, rodents and their ectoparasites. Reports of *R. asembonensis* from arthropods in the country are recent (34, 94, 95) (Supplementary Table 3; Figures 8, 13D), and little is currently known about the biology and potential disease capacity associated with this rickettsia in Brazil.

It was not possible to establish the situation in areas in PR where cases occurred with hospitalizations, but without deaths (Figures 6, 7), or even gain a base-line understanding of such scenarios. Although in such areas we detected the presence of ticks (*A. aureolatum, A. ovale, A. parkeri, A. sculptum*, and *R. sanguineus* s.l.) already known to transmit SFR bioagents (e.g., *R. rickettsii, R. parkeri* strain Atlantic Rainforest and *Ca*. R. paranaensis), we do not know if hospitalizations were due to inherent patient conditions – e.g., comorbidities, genetic characteristics, low immunity - or were specific to the pathogen – e.g., rickettsia species not yet implicated in SFR cases in Brazil, or already implicated, but with different pathogenic profile. Additionally, it is possible to hypothesize that those SFR hospitalizations received correct treatment which thus prevented fatal outcomes. Lack of knowledge of such SFR epidemic scenarios is also not uncommon in other areas of the country (9, 34, 36, 48, 49, 96), and is probably associated with the lack of adequate and timely investigations of patients and in the associated environment, that would allow the conditioning factors of each case to be characterized and defined.

Collection of ticks *Amblyomma incisum* and *Amblyomma varium*; the flea *Pulex irritans*; the mites *Androlaelaps rotundus, Gigantolaelaps* sp. and *Gigantolaelaps wolffsohni*; and the louse *Haematopinus suis* during surveillance and investigation of SFR cases constitute isolated records and, according to the literature, these arthropods do not participate in the SFR epidemic cycles (8). Likewise, despite the detection of specimens of *A. longirostre* and *Ixodes* sp. infected with *Rickettsia* sp. and *Rickettsia* SFG, respectively, they appear not to participate in the SFR cycles, but may signal the presence of rickettsial circulation in a region (Supplementary Table 3).

All analyzed scenarios from PR show eco-epidemiological aspects strongly-associated with outbreaks (Norte Pioneiro, metropolitan, coastal and southern regions), with cases varying clinically across the sample area, illustrating the complexity of the SFR regional profile (81, 97). SFR remains a challenge for the Brazilian public health system, due to a lack of knowledge among health professionals of its clinical characteristics, and eco-epidemiological aspects associated with infection risk (exposure to potential vector ticks and transmission areas), thus likely resulting in underreporting (1, 9). This indicates there is still much to be studied and understood regarding the SFR situation in PR.

Although this study sought a better understanding of the dynamics and vulnerability factors for SFR in PR, and has shown the spatial distribution of endemic areas and highlighted outbreak eco-epidemiological aspects, it is suggested that a number of issues should be addressed to generate an effectively focused capacity to prevent and control such cases: (1) training of health professionals to diagnose suspected cases early; (2) identification of new transmission areas; (3) the need for the National Network for Environmental Monitoring for Spotted Fever and other Rickettsial Diseases to investigate vectors in municipalities with confirmed cases; (4) georeferencing of cases (both suspected and confirmed); (5) improvements in vector collection techniques; (6) full completion of investigation form; (7) permanent health education strategies, to make the general population aware of the inherent risks; (8) additional studies in the vulnerable areas of Norte Pioneiro, metropolitan, coastal and southern regions of the state of PR.

## Data Availability Statement

The datasets presented in this study can be found in online repositories. The names of the repository/repositories and accession number(s) can be found in the article/Supplementary Material.

## Ethics Statement

Ethical review and approval was not required for the study on human participants in accordance with the local legislation and institutional requirements. Written informed consent for participation was not required for this study in accordance with the national legislation and the institutional requirements.

## Author Contributions

LD, KB, FR, and GG contributed to the concept, design, and statistical analysis of the work. LD, KB, and GG with the taxonomic identification of ectoparasites. LD and KB contributed to PCR techniques for Rickettsial identification in arthropods. KB with sequencing and molecular analysis of rickettsiae. GG with acquisition of the data. MN in making the maps. KB, FR, GG, EN, and LD with analysis, interpretation, and the drafting the work. All authors contributed revising it critically for important intellectual content, final approval of the version to be published, and agreement to be accountable for all aspects of the work in ensuring that questions related to the accuracy or integrity of any part of the work are appropriately investigated and resolved.

## Conflict of Interest

The authors declare that the research was conducted in the absence of any commercial or financial relationships that could be construed as a potential conflict of interest.

## Supplementary Material

Supplementary Table 1 Data deposit in the Coleção de Artrópodes Vetores Ápteros de Importância em Saúde das Comunidades (CAVAISC; Collection of Wingless Arthropod Vectors of Community Health Importance), Fundação Oswaldo Cruz, of reference specimens of fleas and ticks collected in Paraná state, Brazil.

Supplementary Table 2 Sociodemographic and assistance data and cases of Spotted Fever Rickettsioses by health regions of Paraná state-Brazil, 2006–2017.

Supplementary Table 3 Data from ticks and fleas samples, collected from 2013 to 2018 in state of Paraná-Brazil, submitted to the detection and identification of rickettsiae.

## Abbreviations

(with Portuguese given where acronym used unchanged in text)
SFR: Spotted Fever Rickettsioses
SFG: Spotted Fever Group
PR: Paraná
HR: Health Regions
BSF: Brazilian Spotted Fever
SP: São Paulo
SINAN (*Sistema de Informação de Agravos de Notificação*): Notifiable Diseases Information System
PIL: Probable Infection Location
ESF (*Estratégia de Saúde da Fam*í*lia*): Family Health Strategy
SUS (*Sistema Único de Saúde*): Integrated Health System
DATASUS (*Departamento de Informática do Sistema Único de Saúde*): Integrated Heatlh System Information Department
LIRN: Laboratory of the National Reference of Rickettsial Vectors
FIOCRUZ: Oswaldo Cruz Foundation
CAVAISC (*Coleção de Artrópodes Vetores Ápteros de Importância em Saúde das Comunidades*): Collection of Wingless Arthropod Vectors of Community Health Importance
hA: Haplotype A
hB: Haplotype B.

## References

[1] Parola P Paddock CD Socolovschi C Labruna MB Mediannikov O Kernif T. Update on tick-borne rickettsioses around the world: a geographic approach. Clin Microbiol Rev. (2013) 4:657–702. 10.1128/CMR.00032-13 24092850 PMC3811236

[2] Szabó MPJ Pinter A Labruna MB. Ecology, biology and distribution of spotted-fever tick vectors in Brazil. Front Cell Infect Microbiol. (2013) 3:1–9. 10.3389/fcimb.2013.00027 23875178 PMC3709097

[3] Eremeeva ME Dasch GA. Challenges posed by tick-borne rickettsiae: eco-epidemiology and public health implications. Front Public Health. (2015) 3:55. 10.3389/fpubh.2015.00055 25954738 PMC4404743

[4] Weck B Dall'Agnol B Souza U Webster A Stenzel B Klafke G. Spotted fever group Rickettsia in the Pampa biome, Brazil, 2015-2016. Emerg Infect Dis. (2016) 22:2014–6. 10.3201/eid2211.160859 27767913 PMC5088030

[5] Fang R Blanton LS Walker DH. Rickettsiae as emerging infectious agents. Clin Lab Med. (2017) 37:382–400. 10.1016/j.cll.2017.01.009 28457356

[6] Dumler J Stephen WDH. Rickettsiales. In: Brenner Don SJT Krieg NJ editors. Systematic Bacteriology. Michigan: Springer. (2005) p. 96–114.

[7] Labruna MB Mattar S Nava S Bermudez S Venzal JM Dolz G. Ricketsioses in Latin America, Caribbean, Spain and Portugal. Rev MVZ Cordoba. (2011) 16:2435–57. 10.21897/rmvz.282

[8] Szabó MPJ Nieri-Bastos FA Spolidorio MG Martins TF Barbieri AM Labruna M. *In vitro* isolation from *Amblyomma ovale* (Acari: Ixodidae) and ecological aspects of the Atlantic Rainforest *Rickettsia*, the causative agent of a novel spotted fever rickettsiosis in Brazil. Parasitology. (2013) 140:719–28. 10.1017/S0031182012002065 23363571

[9] Oliveira SV Guimarães JN Reckziegel GC Neves da BMC Araújo-Vilges de KM Fonseca LX. An update on the epidemiological situation of spotted fever in Brazil. J Venom Anim Toxins Incl Trop Dis. (2016) 7:65–72. 10.1186/s40409-016-0077-4 27555867 PMC4994305

[10] Ministério da Saúde do Brasil. Portaria no 1.943 - Doenças de Notificação Compulsória. (2001). Available online at: http://scielo.iec.gov.br/pdf/iesus/v10n1/v10n1a07.pdf. (accessed May 2, 2019).

[11] Ministério da Saúde do Brasil. Portaria n. 1.271 - Doenças de Notificação Compulsória e Imediata. (2014). Available online at: http://bvsms.saude.gov.br/bvs/saudelegis/gm/2014/prt1271_06_06_2014.html (accessed Mar 9, 2020).

[12] Ministério da Saúde do Brasil. Sistema de Informação de Agravos de Notificação - Sinan. Secretaria de Vigilância em Saúde. (2019). Available online at: https://portalarquivos2.saude.gov.br/images/pdf/2019/junho/14/Casos-de-Febre-Maculosa.pdf (accessed June 2, 2020).

[13] Ministério da Saúde do Brasil. Óbitos de febre maculosa. Brasil, Grandes Regiões e Unidades Federadas. 2000-2019. (2019). Available online at: https://www.saude.gov.br/images/pdf/2019/junho/14/Obitos-por-febre-maculosa.pdf (accessed June 2, 2020).

[14] Governo do Estado do Paraná. Nota Técnica n° 001/2019. Secretaria de Estado da Saúde do Paraná: relatório de situação: Paraná. Secretaria de Estado da Saúde do Paraná. Divisão de Doenças Transmitidas por Vetores. Coordenadoria de Vigilância Ambiental. Diretoria de Atenção e Vigilância em Saúde. (2019). Available online at: http://www.saude.pr.gov.br/arquivos/File/NT_001_FebreMaculosa.pdf (accessed June 2, 2020).

[15] Instituto Brasileiro de Geografia e Estatística (2020). Available online at: https://www.ibge.gov.br/ (accessed March 9, 2020).

[16] Ministério da Saúde do Brasil. Secretaria de Vigilância em Saúde. Guia de Vigilância em Saúde. Febre maculosa brasileira e outras riquetsioses. (2017). Available online at: https://portalarquivos.saude.gov.br/images/pdf/2017/outubro/06/Volume-Unico-2017.pdf (accessed June 2, 2020).

[17] Ministério da Saúde do Brasil. Ficha de investigação: febre maculosa. Sistema de Informação de Agravos de Notificação. (2020). Available online at: https://portalsinan.saude.gov.br/images/documentos/Agravos/Febre%20Maculosa/Febre_Maculosa_v5.pdf (accessed June 2, 2020).

[18] Ministério da Saúde do Brasil. Resolução n. 466, de 12 de dezembro de 2012. Sobre diretrizes e normas regulamentadoras de pesquisas envolvendo seres humanos. Conselho Nacional de Saúde. (2012). Available online at: http://bvsms.saude.gov.br/bvs/saudelegis/cns/2013/res0466_12_ 12_2012.html (accessed June 2, 2020).

[19] Oliveira SV Pereira SVC Barros e Silva PMR Pereira JM Gomes V Amorim M. Vigilância de ambientes da febre maculosa brasileira e outras riquetsioses: a etapa inicial de uma proposta para a formação de rede. Rev Pan Amaz Saúde. (2015) 6:67–71. 10.5123/S2176-62232015000300009

[20] Ferris G. The sucking lice. Mem Pac Coast Entomol Soc. (1951) 1:1–320. 10.5962/bhl.title.149669

[21] Furman DP. *Laelapid mites* (Laelapidae: Laelapinae) of Venezuela. Brigham Young Univ Sci Bull Biol Ser. (1972) 27:1–58.

[22] Bicho CL Ribeiro PB. Chave Pictórica para as principais espécies de *Siphonaptera* de importância médica e veterinária no Brasil. Rev Bras Parasitol Vet. (1998) 7:47–51.

[23] Linardi PM Guimarães LR. Sifonápteros do Brasil. São Paulo: Museu de Zoologia da Universidade de São Paulo (2000).

[24] Price R Hellenthal R Palma R Johnson K Clayton D. The Chewing Lice: World Checklist and Biological Overview. 1st ed. Champaign. IL: Illinois Natural History Survey Special Publication (2004).

[25] Krantz GW Walter DEA. Manual of Acarology. 3rd ed. Lubbock: Texas Tech University Press (2009).

[26] Dantas-Torres F Martins TF Munoz-Leal S Onofrio VC Barros-Battesti DM. Ticks (Ixodida: Argasidae, Ixodidae) of Brazil: Update species checklist and taxonomic keys. Ticks and Tick Borne Dis. (2019) 10:101252. 10.1016/j.ttbdis.2019.06.012 31255534

[27] Aljanabi SM. Martinez I. Universal and rapid salt-extraction of high quality genomic DNA for PCR- based techniques. Nucl Acids Res. (1997) 25:4692–93. 10.1093/nar/25.22.4692 9358185 PMC147078

[28] Labruna MB Whitworth T Horta MC Bouyer DH Mcbride JW Pinter A. *Rickettsia* species infecting *Amblyomma cooperi* ticks from an area in the state of São Paulo, Brazil, where Brazilian spotted fever is endemic. J Clin Microbiol. (2004) 42:90–8. 10.1128/JCM.42.1.90-98.2004 14715737 PMC321730

[29] Labruna MB McBride JW Bouyer DH Camargo LMA Camargo EP Walker DH. Molecular evidence for a spotted fever group *Rickettsia* species in the tick *Amblyomma longirostre* in Brazil. J Med Entomol. (2004) 41:533–7. 10.1603/0022-2585-41.3.533 15185961

[30] Regnery RL Spruill CL Plikaytis BD. Genotypic identification of Rickettsiae and estimation of intraspecies sequence divergence for portions of two rickettsial genes. J Bacteriol. (1991) 173:1576–89. 10.1128/JB.173.5.1576-1589.1991 1671856 PMC207306

[31] Webb L Mitchell C Malloy DC Dasch GA Azad AF. Detection of murine typhus infection in fleas by using the polymerase chain reaction. J Clin Microbiol. (1990) 28:530–4. 10.1128/JCM.28.3.530-534.1990 2108995 PMC269657

[32] Roux V Raoult D. Phylogenetic analysis of members of the genus *Rickettsia* using the gene encoding the outer-membrane protein rOmpB (OmpB). Internat J Syst Evol Microbiol. (2000) 50:1449–55. 10.1099/00207713-50-4-1449 10939649

[33] Bitencourth K. Amblyomma sculptum Berlese 1888, Amblyomma ovale Koch 1844 e Amblyomma aureolatum (Pallas 1772) (Acari: Ixodidae): diversidade genética e detecção de riquétsias em diferentes biomas do Brasil (Master's thesis). Fundação Oswaldo Cruz, Rio de Janeiro, RJ (2017).

[34] Bitencourth K Amorim M de Oliveira SV Voloch CM Gazêta GS. Genetic diversity, population structure and rickettsias in *Amblyomma ovale* in areas of epidemiological interest for spotted fever in Brazil. Med Vet Entomol. (2019) 33:256–68. 10.1111/mve.12363 30746741

[35] Borsoi ABP Bitencourth K de Oliveira SV Amorim M Gazêta GS. Human parasitism by *Amblyomma parkeri* ticks infected with *Candidatus* Rickettsia paranaensis, Brazil. Emerg Infect Dis. (2019) 25:2339. 10.3201/eid2512.190988 31742531 PMC6874247

[36] Sato TP Moura-Martiniano NO Vizzoni VF Silva AB Oliveira SV Amorim M. *Rhipicephalus* (*Boophilus*) *microplus*: Rickettsiae infection in Brazil. Int J of Acarol. (2020) 46:88–93. 10.1080/01647954.2020.1720289

[37] Thompson JD Higgins DG Gibson TJ. CLUSTAL W: improving the sensitivity of progressive multiple sequence alignment through sequence weighting, position-specific gap penalties and weight matrix choice. Nucleic Acids Res. (1994) 22:4673–80. 10.1093/nar/22.22.4673 7984417 PMC308517

[38] Guindon S Dufayard JF Lefort V Anisimova M Hordijk W Gascuel O. New algorithms and methods to estimate maximum-likelihood phylogenies: assessing the performance of PhyML 3.0. Syst Biol. (2010) 59:307–21. 10.1093/sysbio/syq010 20525638

[39] Tamura K Stecher G Peterson D Filipski A Kumar S. MEGA6: molecular evolutionary genetics analysis version 6.0. Mol Biol Evol. (2013) 30:2725–9. 10.1093/molbev/mst197 24132122 PMC3840312

[40] Anisimova M Gascuel O. Approximate likelihood-ratio test for branches: a fast, accurate, and powerful alternative. Syst Biol. (2006) 55:539–52. 10.1080/10635150600755453 16785212

[41] Pinter A Costa CS Holcman MM Camara M Leite MM. A Febre Maculosa Brasileira na Região Metropolitana de São Paulo. BEPA. (2016) 13:1–45.

[42] Montenegro DC Bitencourth K de Oliveira SV Borsoi AP Cardoso KM Sousa MSB. Spotted fever: epidemiology and vector-rickettsia-host relationship in Rio de Janeiro state. Front Microbiol. (2017) 8:505. 10.3389/fmicb.2017.00505 28424664 PMC5371726

[43] Valente MD Silva PW Arzua M Barros-Battesti DM Martins TF Silva AM. Records of ticks (Acari: Ixodidae) on humans and distribution of spotted-fever cases and its tick vectors in Paraná State, southern Brazil. Ticks Tick Borne Dis. (2020) 11:101510. 10.1016/j.ttbdis.2020.101510 32993930

[44] Nasser JT Lana RC Silva CMDS Lourenço RW Silva DCDCE Donalísio MR. Urbanization of Brazilian spotted fever in a municipality of the southeastern region: epidemiology and spatial distribution. Rev Bras Epidemiol. (2015) 18:299–312. 10.1590/1980-5497201500020002 26083504

[45] Souza CE Pinter A Donalisio MR. Risk factors associated with the transmission of Brazilian spotted fever in the Piracicaba river basin, State of São Paulo, Brazil. Rev Soc Bras Med Trop. (2015) 48:11–7. 10.1590/0037-8682-0281-2014 25860458

[46] Krawczak FS Muñoz-Leal S Guztzazky AC Oliveira SV Santos FCP Angerami RN. Case report: *Rickettsia* sp. strain atlantic rainforest infection in a patient from a spotted fever-endemic area in southern Brazil. Am J Trop Med Hyg. (2016) 95:551–3. 10.4269/ajtmh.16-0192 27325804 PMC5014257

[47] Moerbeck L Vizzoni VF Machado-Ferreira E Cavalcante RC Oliveira SV Soares CAG. *Rickettsia* (Rickettsiales: Rickettsiaceae) vector biodiversity in high altitude Atlantic forest fragments within a semiarid climate: a new endemic area of spotted-fever in Brazil. J Med Entomol. (2016) 53:1458–66. 10.1093/jme/tjw121 27480099

[48] Bitencourth K Amorim M Oliveira SV Caetano RL Voloch CM Gazêta GS. *Amblyomma sculptum*: genetic diversity and rickettsias in the Brazilian Cerrado biome. Med Vet Entomol. (2017) 31:427–37. 10.1111/mve.12249 28752684

[49] Oliveira SV Willemann MCA Gazêta GS Angerami RN Gurgel-Gonçalves R. Predictive factors for fatal tick-borne spotted fever in Brazil. Zoonoses Public Health. (2017) 64:44–50. 10.1111/zph.12345 28169507

[50] Machado IB Bitencourth K Cardoso KM Oliveira SV Santalucia M Marques SFF. Diversity of rickettsiae and potential vectors of spotted fever in an area of epidemiological interest in the Cerrado biome, midwestern Brazil. Med Vet Entomol. (2018) 32:481–9. 10.1111/mve.12315 29972600

[51] Batista FG Silva DM Green KT Tezza LBL Vasconcelos SP Carvalho SGS. Serological survey of *Rickettsia* sp. in horses and dogs in an non-endemic area in Brazil. Rev Bras Parasitol Vet. (2010) 19:205–9. 10.1590/S1984-29612010000400003 21184695

[52] Fortes FS Silveira I Moraes-Filho J Leite RV Bonacim JE Biondo AW. Seroprevalence of *Rickettsia bellii* and *Rickettsia felis* in dogs, São José dos Pinhais, State of Paraná, Brazil. Rev Bras Parasitol Vet. (2010) 19:222–27. 10.1590/S1984-29612010000400006 21184698

[53] Freitas MC Grycajuk M Molento MB Bonacin J Labruna MB Pacheco RC. Brazilian spotted fever in cart horses in a non-endemic area in Southern Brazil. Rev Bras Parasitol Vet. (2010) 19:130–1. 10.1590/S1984-29612010000200013 20624353

[54] Otomura FH Sangioni LA Pacheco RC Labruna MB Galhardo JA Ribeiro MG. Anticorpos anti-rickettsias do grupo da febre maculosa em equídeos e caninos no norte do Paraná, Brasil. Arq Bras Med Vet Zootec. (2010) 62:761–64. 10.1590/S0102-09352010000300037

[55] Tamekuni K Toledo RS Silva Filho MF Haydu VB Pacheco RC Cavicchioli JH. Serosurvey of antibodies against spotted fever group *Rickettsia* spp. in horse farms in Northern Paraná, Brazil. Rev Bras Parasitol Vet. (2010) 19:1–3. 10.1590/S1984-29612010000400014 21184706

[56] Fortes FS Santos LC Cubas ZS Barros-Filho IR Biondo AW Silveira I. Anti-*Rickettsia* spp. antibodies in free-ranging and captive capybaras from southern Brazil. Pesq Vet Bras. (2011) 31:1014–8. 10.1590/S0100-736X2011001100013

[57] Tamekuni K Toledo RS Silva Filho MF Haydu VB Pacheco RC Labruna MB. Survey of rickettsiae in humans, dogs, horses and ticks in Northen Paraná, Brazil. Semina Ciências Agrárias. (2011) 32:1527–38. 10.5433/1679-0359.2011v32n4p1527

[58] Toledo RS Tamekuni K Silva Filho MF Haydu VB Barbieri AR Hiltel AC. Infection by spotted fever rickettsiae in people, dogs, horses and ticks in Londrina, Parana State, Brazil. Zoonoses Public Health. (2011) 58:416–23. 10.1111/j.1863-2378.2010.01382.x 21824336

[59] Pacheco RC Arzua M Nieri-bastos FA Moraes-filho J Marcili A Richtzenhain LJ. Rickettsial infection in ticks (Acari: Ixodidae) collected on birds in southern Brazil. J Med Entomol. (2012) 49:710–6. 10.1603/ME11217 22679880

[60] Otomura FH Truppel JH Moraes Filho J Labruna MB Rossoni DF Massafera R. Probability of occurrence of the Brazilian spotted fever in northeast of Paraná state, Brazil. Ver Bras Parasitol Vet. (2016) 25:394–400. 10.1590/s1984-29612016060 27925056

[61] Galvão MAM. Febre maculosa em Minas Gerais: um estudo sobre a distribuição da doença no Estado e seu comportamento em área de foco peri-urbano (Dissertation/master's thesis). Universidade Federal de Minas Gerais, Belo Horizonte, MG (1996).

[62] Lemos ERS Machado RD Coura JR Guimarães MAA Serra-Freire NM Amorim M. Epidemiological aspects of the brazilian spotted fever: seasonal activity of ticks collected in an endemic area in São Paulo, Brazil. Rev Soc Bras Med Trop. (1997) 30:181–85. 10.1590/S0037-86821997000300002 9197151

[63] Labruna MB Kasai N Ferreira F Faccini JL Gennari SM. Seasonal dynamics of ticks (Acari: Ixodidae) on horses in the state of São Paulo, Brazil. Vet Parasitol. (2002) 105:65–77. 10.1016/S0304-4017(01)00649-5 11879967

[64] Pinter A França AC Souza CED Sabbo C Nascimento EMMD Santos FCPD. Febre Maculosa Brasileira. Bepa Boletim Epidemiol Paulista. (2011) 8:1–32.

[65] Szabó MPJ Pereira LDF Castro MB Garcia MV Sanches GS Labruna MB. Biology and life cycle of *Amblyomma incisum* (Acari: Ixodidae). Exp Appl Acarol. (2009) 48:263–71. 10.1007/s10493-008-9234-y 19130270

[66] Suzin A Vogliotti A Nunes PH Barbieri ARM Labruna MB Szabó MPJ. Free-living ticks (Acari: Ixodidae) in the Iguaçu National Park, Brazil: temporal dynamics and questing behavior on vegetation. Ticks Tick Borne Dis. (2020) 11:101471. 10.1016/j.ttbdis.2020.101471 32723660

[67] Angerami RN Resende MR Feltrin AFC Katz G Nascimento EM Stucchl RSB. Brazilian spotted fever: a case series from an endemic area in Southeastern Brazil epidemiological aspects. Ann NY Acad Sci. (2006) 1078:170–2. 10.1196/annals.1374.030 17114702

[68] Seyferth G. Imigração, colonização e identidade étnica (notas sobre a emergência da etnicidade em grupos de origem européia no Sul do Brasil). Rev de Antropol. (1986) 29:1986. 10.2307/41601919

[69] Lemos ER Alvarenga FB Cintra ML Ramos MC Paddock CD. Spotted fever in Brazil: a seroepidemiological study and description of clinical cases in an endemic area in the state of Sao Paulo. Am J Trop Med Hyg. (2001) 65:329–34. 10.4269/ajtmh.2001.65.329 11693878

[70] Lemos E Rozental T Villela CL. Brazilian spotted fever: description of a fatal clinical case in the state of Rio de Janeiro Febre Maculosa Brasileira: descrição de um caso fatal no Estado do Rio de Janeiro. Rev Soc Bras Med Trop. (2002) 35:523–5. 10.1590/S0037-86822002000500017 12621675

[71] Secretaria do Estado da Saúde São Paulo. Centro de controle de Doenças. Febre Maculosa Brasileira. Bol Epidemiol Paulo. Vol. 8 (2011) (suppl I). Available online at: http://www.saude.sp.gov.br/resources/sucen/homepage/downloads/arquivos-de-febre-maculosa/bepa94_suplemento_fmb.pdf (accessed February 20, 2014).

[72] Martins TF Barbieri AR Costa FB Terassini FA Camargo LM Peterka CR. Geographical distribution of *Amblyomma cajennense* (sensu lato) ticks (Parasitiformes: Ixodidae) in Brazil, with description of the nymph of *A. cajennense* (sensu stricto). Parasites Vectors. (2016) 9:1–14. 10.1186/s13071-016-1460-2 27036324 PMC4818509

[73] Ueno TEH Costa FB Moraes-Filho J Agostinho WC Fernandes WR. Experimental infection of horses with *Rickettsia rickettsii*. Parasites Vectors. (2016) 9:1–11. 10.1186/s13071-016-1784-y 27624315 PMC5022194

[74] Pinter A Labruna MB. Isolation of *Rickettsia rickettsii* and *Rickettsia bellii* in cell culture from the tick *Amblyomma aureolatum* in Brazil. Ann NY Acad Sci. (2006) 1078:523–9. 10.1196/annals.1374.103 17114770

[75] Piranda EM Faccini JL Pinter A Pacheco RC Cançado PH Labruna MB. Experimental infection of *Rhipicephalus sanguineus* ticks with the bacterium *Rickettsia rickettsii*, using experimentally infected dogs. Vector Borne Zoonotic Dis. (2011) 11:29–36. 10.1089/vbz.2009.0250 20569011

[76] Gehrke FS Gazeta GS Souza ER Ribeiro A Marrelli MT Schumaker TT. *Rickettsia rickettsii, Rickettsia felis* and *Rickettsia* sp. TwKM03 infecting *Rhipicephalus sanguineus* and *Ctenocephalides felis* collected from dogs in a Brazilian spotted fever focus in the State of Rio de Janeiro/Brazil. Clin Microbiol Infec. (2009) 15:267–8. 10.1111/j.1469-0691.2008.02229.x 19298400

[77] De Almeida RFC Garcia MV Cunha RC Matias J e Silva EA Matos MDFC. Ixodid fauna and zoonotic agents in ticks from dogs: first report of *Rickettsia rickettsii* in *Rhipicephalus sanguineus* in the state of Mato Grosso do Sul, mid-western Brazil. Exp Appl Acarol. (2013) 60:63–72. 10.1007/s10493-012-9641-y 23229491

[78] Silva AB Duarte MM Costa Cavalcante R Oliveira SV Vizzoni VF. *Rickettsia rickettsii* infecting *Rhipicephalus sanguineus* sensu lato (Latreille 1806), in high altitude Atlantic Forest fragments, Ceará state, Brazil. Acta Tropica. (2017) 173:30–3. 10.1016/j.actatropica.2017.05.018 28535905

[79] Macaluso KR Sonenshine DE Ceraul SM Azad AF. Rickettsial infection in *Dermacentor variabilis* (Acari: Ixodidae) inhibits transovarial transmission of a second Rickettsia. J Med Entomol. (2002) 39:809–13. 10.1603/0022-2585-39.6.809 12495176

[80] Sangioni LA Horta MC Vianna MCB Gennari SM Soares RM Galvão MAM. Rickettsial infection in animals and Brazilian spotted fever endemicity. Emerg Infect Dis. (2005) 11:265–70. 10.3201/eid1102.040656 15752445 PMC3320454

[81] Oliveira SV Caldas EP Colombo S Gazêta GS Labruna MB Santos FCP. A fatal case of Brazilian spotted fever in a non-endemic area in Brazil: the importance of having health professionals who understand the disease and its areas of transmission. Rev Soc Bras Med Trop. (2016) 49:653–5. 10.1590/0037-8682-0088-2016 27812666

[82] Sakai RK Costa FB Ueno TE Ramirez DG Soares JF Fonseca AH. Experimental infection with *Rickettsia rickettsii* in an *Amblyomma dubitatum* tick colony, naturally infected by *Rickettsia bellii*. Ticks Tick Borne Dis. (2014) 5:917–23. 10.1016/j.ttbdis.2014.07.003 25108783

[83] Paddock CD Amy MD Dryden MW Noden BH Lash RR Abdelghani SS. High prevalence of “*Candidatus* Rickettsia andeanae” and apparent exclusion of *Rickettsia parkeri* in adult *Amblyomma maculatum* (Acari: Ixodidae) from Kansas and Oklahoma. Ticks Tick Borne Dis. (2015) 6:297–302. 10.1016/j.ttbdis.2015.02.001 25773931 PMC4487539

[84] Price WH. The epidemiology of Rocky Mountain spotted fever: studies of the biological survival mechanisms of *Rickettsia rickettsii*. Am J Trop Med Hyg. (1954) 60:292–319. 10.1093/oxfordjournals.aje.a119723 13207101

[85] Loving SM Smith AB Disalvo AF Burgdorfer W. Distribution and prevalence of spotted fever group Rickettsiae in ticks from South Carolina, with epidemiological survey of persons bitten by infected ticks. Am J Trop Med Hyg. (1978) 27:1255–60. 10.4269/ajtmh.1978.27.1255 103448

[86] Burgdorfer W Hayes S Thomas LJR Lancaster JL. A new spotted fever group *Rickettsia* from the lone star tick, *Amblyomma americanum*. In: Burgdorfer W Anacker RL editors. Rickettsiae and Rickettsial Diseases. New York, NY: Academic Press (1981). p. 595–602.

[87] Vizzoni VF Silva AB Cardoso KM Santos FB Stenzel B Amorin M. Genetic identification of *Rickettsia* sp. strain Atlantic rainforest in an endemic area of a mild spotted fever in Rio Grande do Sul state, Southern Brazil. Acta Tropica. (2016) 162:142–5. 10.1016/j.actatropica.2016.06.018 27338183

[88] Moraes-Filho J Pinter A Pacheco RC Gutmann TB Barbosa SO Gonzáles MA. New epidemiological data on Brazilian spotted fever in an endemic area of the State of Sao Paulo, Brazil. Vector Borne Zoonotic Dis. (2009) 9:73–8. 10.1089/vbz.2007.0227 18847319

[89] Silva ME Ribeiro RR Costa JO Moraes-Filho J Pacheco RC Labruna MB. Prevalência de anticorpos anti-Rickettsia spp. em cães da cidade de Belo Horizonte, MG. Arq Bras Med Vet Zootec. (2010) 62:1007–10. 10.1590/S0102-09352010000400036

[90] Rozental T Ferreira MS Gomes R Costa CM Barbosa PRA Bezerra IO. A cluster of *Rickettsia rickettsii* infection at an animal shelter in an urban area of Brazil. Epidemiol Infect. (2015) 143:2446–50. 10.1017/S0950268814003161 25483025 PMC9150930

[91] Labruna MB Walker DH. *Rickettsia felis* and changing paradigms about pathogenic rickettsiae. Emerg Infect Dis. (2014) 20:1768. 10.3201/eid2010.131797 25271441 PMC4193273

[92] Blanton LS Walker DH. Flea-borne rickettsioses and rickettsiae. Am J Trop Med Hyg. (2017) 96:53–6. 10.4269/ajtmh.16-0537 27799640 PMC5239709

[93] Raoult D La Scola B Enea M Fournier PE Roux V Fenollar F. A flea-associated *Rickettsia* pathogenic for humans. J Emerg Infect Dis. (2001) 7:73–81. 10.3201/eid0701.010112 11266297 PMC2631683

[94] Dall'Agnol BS Webster U Weck A Weck B Stenzel B Labruna MB. “Candidatus Rickettsia asemboensis” in *Rhipicephalus sanguineus* ticks, Brazil. Acta Trop. (2017) 167:18–20. 10.1016/j.actatropica.2016.12.008 27986544

[95] Silva AB Vizzoni VF Costa AP Costa FB Moraes-Filho J Labruna MB. First report of a *Rickettsia asembonensis* related infecting fleas in Brazil. Acta Trop. (2017) 172:44–9. 10.1016/j.actatropica.2017.04.004 28427962

[96] Oliveira SV Costa RF Ferreira G Pereira SVC Amorim M Monteiro MFM. Fatal case of spotted fever in a patient from Northeastern Brazil. Rev Inst Med Trop São Paulo. (2018) 60:e21. 10.1590/s1678-9946201860021 29846472 PMC5975566

[97] Silveira I Martins TF Olegário MM Peterka C Guedes E Ferreira F. Rickettsial infection in animals, humans and ticks in Paulicéia, Brazil. Zoonoses Public Health. (2015) 62:525–33. 10.1111/zph.12180 25643912

